# GPR124 facilitates pericyte polarization and migration by regulating the formation of filopodia during ischemic injury

**DOI:** 10.7150/thno.34168

**Published:** 2019-08-14

**Authors:** Dan-Yang Chen, Ning-He Sun, Ya-Ping Lu, Ling-Juan Hong, Tian-Tian Cui, Cheng-Kun Wang, Xing-Hui Chen, Shuai-Shuai Wang, Li-Li Feng, Wei-Xing Shi, Kohji Fukunaga, Zhong Chen, Ying-Mei Lu, Feng Han

**Affiliations:** 1 Institute of Pharmacology and Toxicology, College of Pharmaceutical Sciences, Zhejiang University, Hangzhou, 310058, China; 2 Key Laboratory of Cardiovascular & Cerebrovascular Medicine, School of Pharmacy, Nanjing Medical University, Nanjing, 211166, China; 3 Department of Physiology, Nanjing Medical University, Nanjing, 211166, China; 4 School of Medicine, Zhejiang University City College, Hangzhou, 310015, China; 5 Departments of Pharmaceutical, Administrative, and Basic Sciences, Schools of Pharmacy and Medicine, Loma Linda University Health, CA, 92350, USA; 6 Department of Pharmacology, Graduate School of Pharmaceutical Sciences, Tohoku University, Sendai, 980-8578, Japan

**Keywords:** GPR124, focal adhesions, pericytes, directional migration, filopodia, ischemia.

## Abstract

Prolonged occlusion of multiple microvessels causes microvascular injury. G protein-coupled receptor 124 (GPR124) has been reported to be required for maintaining central nervous system (CNS) angiogenesis and blood-brain barrier integrity. However, the molecular mechanisms by which GPR124 regulates pericytes during ischemia have remained elusive.

**Methods**: A microsphere embolism-induced ischemia model was used to evaluate the expression of GPR124 following microsphere embolism. Immunocytochemistry and stochastic optical reconstruction microscopy imaging were used to assess the expression and distribution of GPR124 in human brain vascular pericytes (HBVPs) and after the treatment with 3-morpholino-sydnonimine (SIN-1) or oxygen-glucose deprivation (OGD). The effect of GPR124 knockdown or overexpression on HBVP migration was analyzed in vitro using wound healing assays and a microfluidic device. GPR124 loss-of-function studies were performed in HBVPs and HEK293 cells using CRISPR-Cas9-mediated gene deletion. Time-lapse imaging was used to assess dynamic changes in the formation of filopodia in an individual cell. Finally, to explore the functional domains required for GPR124 activity, deletion mutants were constructed for each of the N-terminal domains.

**Results**: GPR124 expression was increased in pericytes following microsphere embolism. Morphological analysis showed localization of GPR124 to focal adhesions where GPR124 bound directly to the actin binding protein vinculin and upregulated Cdc42. SIN-1 or OGD treatment redistributed GPR124 to the leading edges of HBVPs where GPR124 signaling was required for pericyte filopodia formation and directional migration. Partial deletion of GPR124 domains decreased SIN-1-induced filopodia formation and cell migration.

**Conclusion**: Taken together, our results provide the first evidence for a role of GPR124 in pericyte migration under ischemic conditions and suggest that GPR124 was essential for Cdc42 activation and filopodia formation.

## Introduction

Continuous flow of blood containing sufficient oxygen and glucose through the microvasculature ensures the brain function and vitality 1. Cerebral microvascular occlusion, a brain pathological phenomenon, is implicated in the induction of stroke 2,3. Cerebral ischemic stroke eventually leads to a loss of neurological function. However, the molecular and cellular mechanisms that link microvascular occlusion to vascular remodeling remain elusive.

GPR124, an important member of the adhesion-type G protein-coupled receptor (aGPCR) family 4,5, is a long-chain protein consists of 1338 amino acids integrating seven classic α-helix transmembrane (TM) domains as well as numerous conserved extracellular N-terminal domains. Its N-terminal region is composed of multiple leucine-rich regions (leucine-rich repeats, LRRs), an immunoglobulin (Ig)-like domain, a hormone-binding domain (Horm) and a GPCR proteolytic site (GPS) 5,6.

GPR124 has been reported to control the central nervous system (CNS) angiogenesis and blood-brain barrier (BBB) integrity. Global or endothelium-specific GPR124 deletion results in embryonic lethality in mice, mainly due to reduced CNS angiogenesis along with the generation of pathological glomeruloid structures and bleeding 7-10. Genetic inactivation of GPR124 in the zebrafish similarly impairs vascularization and BBB maturation 11. Surprisingly, postnatal or adult GPR124 deletion does not affect BBB integrity 12. Recent studies have highlighted some essential yet long ignored roles played by GPR124 in various pathophysiological processes of cerebrocardiovascular diseases 12,13. For instance, GPR124 receptors are involved in the development of high blood pressure 14,15. Furthermore, GPR124 and Reck form a multiprotein complex with the Frizzled receptor and the Lrp5/6 coreceptor that strongly enhances canonical Wnt signaling by Wnt7a and Wnt7b, specifically in CNS endothelial cells (ECs) 11,16,17,18. However, detailed investigation of GPR124 function in pericytes is still lacking.

Pericytes, which are important components of the neurovascular unit 19, form the basic structure of microvessels and maintain the stability of the BBB 20,21. Pericytes perform essential physiological functions in the CNS, such as blood vessel formation and stabilization 22,23 and capillary diameter and cerebral blood flow regulation 24, and are associated with numerous CNS disorders 25,26. In addition, pericytes are critically involved in endothelial angiogenesis. This process could be provoked by various risk factors, followed by the pericyte recruitment, migration and enhancing the maturity and stability of newly formed capillaries 27. However, the role of pericytes in neurovascular unit reconstruction after the cerebral injury is yet to be revealed.

The goal of the study was to understand the role GPR124 plays in pericyte polarization and migration in response to ischemia-like insult. We provide evidence that GPR124 colocalizes with vinculin in pericytes, regulates filopodia formation, and is required for pericyte polarization and migration during ischemia.

## Methods

### Cell culture

Human brain vascular pericytes (HBVPs) were purchased from ScienCell (#1200), and human embryonic kidney 293 (HEK293) cells were purchased from the American Type Culture Collection (ATCC; Cat# CRL-1573). The cells were maintained in Dulbecco's modified Eagle's medium (DMEM, Gibco) supplemented with 10% fetal bovine serum (FBS, Gibco) and 1% penicillin/streptomycin, and cultured at 37 °C in 5% CO_2_. For nitrosative stress studies, cells were treated with 0.5 mM 3-morpholino-sydnonimine hydrochloride (SIN-1, M5793, Sigma-Aldrich) for 12 h.

### Microsphere-induced permanent focal cerebral ischemic model

All animal procedures were approved by the Committee on Animal Experiments at Zhejiang University and were in complete compliance with the National Institutes of Health Guide for the Care and Use of Laboratory Animals. The microsphere embolism (ME) model was prepared as described previously 2,28. Briefly, male mice were anesthetized with 3% isoflurane and maintained in mask filled with O_2_-rich air that contained 1.5% isoflurane. After placing the mouse in the supine position, the external carotid artery (ECA) and the pterygopalatine artery (PPA) were temporarily blocked with aneurysm clips, and then 5000 non-radioactive microspheres (15 μm in diameter) were slowly injected into the left common carotid artery (CCA). The sham group received the same treatment except for the injection of solution free of microspheres. The rectal temperature was monitored and maintained at 37 ± 0.5 °C with a heating blanket throughout the process. The mice were decapitated at 72 h after the injection of microspheres, and the cortical areas of the ipsilateral hemisphere were collected for further analysis.

### Isolation of mouse brain pericytes and endothelial cells

The isolation of mouse brain pericytes (PCs) and endothelial cells (ECs) was performed as described with some modifications 29. In brief, adult mice were killed, with their brains removed and incubated with collagenase/dispase (Sigma-Aldrich, cat. no. 11097113001). Corresponding myelin and debris were eliminated by centrifugation via Percoll. Finally, single-cell suspensions are labeled with magnetic beads (Dynabeads, Invitrogen, #11035) coated with either rat anti-mouse CD13 antibody (BD Pharmingen, #558744) or rat anti-mouse CD31 antibody (BD Pharmingen, #553370). For immunofluorescence assay, the beads were washed with Isolation Buffer (Ca^2+^- and Mg^2+^-free PBS supplemented with 0.1% BSA and 2 mM EDTA, pH = 7.4) and then cells were resuspended in EGM-2 medium (Lonza) and placed in one well of a 6-well plate. For RNA extraction, magnetically enriched cells were immediately lysed for Real-Time PCR assay (RT-PCR).

### Quantitative Real-Time PCR

For detection of mRNA level at 72 h after the injection of microspheres, the cortical areas of the ipsilateral hemisphere were dissected, homogenized, and the total RNAs were isolated using RNAiso Plus (Takara). Corresponding cDNA was synthesized with the PrimeScript RT reagent Kit Perfect Real Time (Takara) according to the manufacturer's instructions. Quantitative Real-Time PCR (qRT-PCR) was performed in 96-well plates by Bio-Rad CFX96 Touch Deep Well Real-Time PCR Detection System (USA) using SYBR Premix Ex Taq (Takara). The PCR primers for GPR124 were (forward 5′-TCACGCTCACCAACTACCAAATG-3′) and (reverse 5′-CCTCCAGCAATCAAGTAGAAC-3′). The PCR primers for CD13 were (forward 5′-GCCCCATGAAGAGGTATCTGAAG-3′) and (reverse 5′-TGCAGTAGACAGTAGACCGAAG-3′). The PCR primers for CD31 were (forward 5′-CTGCCAGTCCGAAAATGGAAC-3′) and (reverse 5′-CTTCATCCACTGGGGCTATC-3′). The Ct values were automatically calculated using CFX ManagerTM software. RNA expression was calculated using the comparative Ct method by normalizing to β-actin.

### Immunoprecipitation

Protein-protein complexes were captured from the whole cell extracts using the Capturem IP & Co-IP Kit (Takara, 635721) following the manufacturer's instructions. Cells grown in 35-mm dishes were washed with PBS and incubated with 600 μl of Lysis/Equilibration Buffer containing protease inhibitors on ice for 15 min. Cell lysates were centrifuged at 17,000 × g for 10 min at 4 °C. 50 μl of total protein sample was transferred to new tubes used as the input. The remaining lysates were incubated with anti-Paxillin (ThermoFisher, MA5-13356), anti-Vinculin (Millipore, MAB3574), and special IgG (Santa, sc-2025) antibodies for 1 h at 4 ℃, respectively. Afterward, the sample was loaded onto the spin column and centrifuged at 1,000 × g for 1 min at room temperature (RT). The spin column was washed twice with 100 μl of Wash Buffer and centrifuged at 1,000× g for 1 min at RT. Immunoprecipitated proteins were eluted with Elution Buffer. The cell total proteins and immunoprecipitated proteins were further subjected to sodium dodecyl sulfate polyacrylamide gel electrophoresis (SDS-PAGE).

### Focal adhesion isolation

HBVPs were plated (24 h, 60% confluence) on 60-mm dishes coated with 10 μg/mL fibronectin. Cells were treated with or without SIN-1 (0.5 mM) for 12 h. Cells were shocked with triethanolamine-containing low-ionic-strength buffer for 6 min at RT (2.5 mM triethanolamine in water at pH 7.0) as described previously 30. Briefly, cell bodies were removed using a Waterpik that pulsed hydrodynamic force with PBS containing protease inhibitors (Roche) throughout the dish. Such a treatment was repeated to ensure the no excess cell bodies existed (10 s for each time of wash with *ca.* 6 mL PBS). The buffer from three dishes was collected after triturating each dish and centrifuged at 1,500 rpm for 4 min at 4 °C, serving as the “cell body fraction” for western blotting. Focal adhesions remaining bound to the dish were rinsed using the Waterpik with 100 mL buffer, collected and denatured by scraping with a rubber policeman in 1×RIPA (radio-immunoprecipitation assay) buffer (50 mM Tris-HCl at pH 8.0, 150 mM NaCl, 1.0% NP-40 and 0.5% sodium deoxycholate) containing 1% SDS, and sonicated for 15 s on ice. Total protein levels were determined by the DC™ Protein Assay (Bio-Rad, 5000116), and these values were exploited to normalize the loading volumes of protein samples.

### Western blotting

Immunoblotting was carried out in different cellular fractions (total cell lysate, isolated focal adhesion fractions and cell body fractions) after determination of protein concentrations using the DC™ Protein Assay (Bio-Rad, 5000116) 31. Briefly, the cell lysates containing equivalent amounts of protein were loaded and separated by SDS-PAGE and immunodetected with antibodies: rabbit anti-GPR124 (TEM5, 1:1,500, ThermoFisher, PA5-20442), mouse anti-Vinculin (1:3,000, Millipore, MAB3574), mouse anti-Flag (1:500, Sigma-Aldrich, F3165), mouse anti- Paxillin (1:3,000, ThermoFisher, MA5-13356), mouse anti-β-actin (1:5,000, Cell Signaling Technology, 3700). After incubation for 12 h at 4 °C, membranes were incubated with the appropriate horseradish peroxidase-conjugated secondary antibody. Immunoreactivity was visualized by enhanced chemiluminescence (Amersham Life Science).

### Plasmid constructs

pEGFP-GPR124 were generated by cloning the entire coding region of the mouse GPR124 gene into the NheI and HindIII sites of the pEGFP-N1 plasmid. The Myc epitope (EQKLISEEDL) was inserted into the C-terminal of GPR124 sequence to generate the GPR124-Myc. Myc-tagged GPR124 was used as a backbone to generate the following GPR124 mutants: ΔLRR (Δ amino acids (Δaa) 37-245), ΔIg (Δaa253-348), ΔHorm (Δaa352-427), ΔGPS (Δaa506-756), ΔExtra (Δaa37-756), ΔPDZ (Δaa1333-1338). In this work, LRRs referred to the entire domain including multiple leucine-rich regions and LRR/CT. Lifeact-EGFP was constructed by cloning the Lifeact gene which was synthesized by Genewiz (China) into the pEGFP-N1 plasmid. All these plasmids were verified by DNA sequencing.

### Lentivirus construction

To knock down GPR124 in HBVPs, a lentiviral vector (pLKD-CMV-eGFP-U6-shRNA) carrying shRNA targeting GPR124 or a control nontargeting vector was constructed. Briefly, GPR124 shRNA (5'-GGAGCTGAAGCGTTTAGATCT-3') or control shRNA (5'-TTCTCCGAACGTGTCACGT-3') was inserted into the lentiviral vector construct and driven by the U6 promoter. In order to construct GPR124-overexpressing HBVPs, pLenti-CMV-GPR124-3Flag was cloned by replacing that of eGFP sequence in the pLenti-CMV-eGFP-3Flag vector.

To knock out GPR124 in HBVPs and HEK293 cells, a 23 nt target DNA sequence (5'-CCTTCTGCCTAACGGCACCGTTA-3') in exon 1 of the human GPR124 genomic locus (NC_000008.11) was selected for the generation of a single guide RNA (sgRNA, 5'-TAACGGTGCCGTTAGGCAGA-3') for SpCas9 using a CRISPR design tool. To construct the SpGuide, the top oligo (5′-caccgTAACGGTGCCGTTAGGCAGA-3′) and bottom oligo (5′-aaacTCTGCCTAACGGCACCGTTAc-3′) were annealed and cloned into the pLenti-U6-sgRNA-CMV-Puro-P2A-3Flag-SpCas9 vector by BbsI. All clones were confirmed by DNA sequencing using the primer 5′-GGACTATCATATGCTTACCG-3′ from the sequence of the U6 promoter, which drove the expression of the sgRNA.

### Lentivirus production and transduction

Lentiviral particles were produced by OBiO Technology (China). For GPR124 shRNA or overexpression lentivirus transduction, HBVPs were transduced with pLKD-CMV-eGFP-U6-shRNA or pLenti-CMV-GPR124-3Flag lentivirus and supplemented with 2.5 μl of 1 mg/mL polybrene (Millipore) in 24-well plates (multiplicity of infection, MOI = 20). For Cas9 lentivirus transduction of the HBVPs or HEK293 cells, puromycin (2 or 0.5 μg/mL, respectively) selection was performed for 3 days. Clonal cell lines were generated by picking up an individual colony.

### Sequencing analysis for genome modification

Cells were collected for genomic DNA extraction using the AxyPrep Multisource Genomic Miniprep Kit (Axygen) by following the manufacturer's protocol. In brief, cells were resuspended in the phosphate-buffered saline (PBS, pH 7.4) solution containing RNaseA, and vortexed for 15 s, followed by the introduction of Proteinase K (vortexed for 60 s) and incubation at 56 °C for 10 min. The genomic region around the target DNA sequence was amplified using PCR with PrimeSTAR® HS DNA Polymerase (TaKaRa). The PCR primers were (forward 5′-CCGCGGGGCGATGGGTTGATGGGC-3′) and (reverse 5′-GTGCCTTTCTCTGGGGTGCGCCTG-3′). The PCR products were separated in 1.5% agarose gel and purified with a AxyPrep DNA Gel Extraction Kit (Axygen) for DNA sequencing. DNA sequencing was performed by Genewiz.

### Wound healing assay

HBVPs were transfected with full-length GPR124 using lentivirus system and HEK293 cells were transfected with GPR124-Myc or various GPR124 truncates with Lipofectamine 3000 Reagent (Invitrogen) for 24 h before seeding into 6-well plates. The cells were expected to reach a 90% confluency on the day of the experiment. Monolayers were scraped using a pipette tip and washed for three times with pre-warmed complete growth medium. The wounded monolayer was imaged using a microscope (Leica) equipped with a 10× objective, and images were captured after 24 h. The relative migration of the cells (%) was evaluated based on the area of the wound edge.

### Live cell imaging

All imaging was performed on a spinning disk confocal microscope (Olympus, DU-897D-CS0) equipped with a 60× 1.4 NA oil immersion objective lens. Cells or GPR124-knockout cells were transfected with Lifeact-EGFP for 24 h before seeding into 35-mm glass-bottom dishes (Nest) and overnight culture. The cells were incubated with serum-free medium for 4 h and then imaged at 37 °C in a serum-free medium supplemented with 20 mM HEPES for a duration of 30 min. After that, cells were treated with SIN-1 (final concentration: 0.5 mM) in the serum-free medium, and the imaging was continued for another 30 min. During this, cells were maintained at 37 °C in a chamber containing 5% CO_2_. After acquisition, the image process with a focus on the number and length of filopodia or filopodia-like cytoskeleton was conducted using the software package MATLAB R2011b (Mathworks, USA).

### HBVPs migration assays

For live migration assay, HBVPs were suspended in a serum-free medium and seeded into the microfluidic device (Ibidi). A 6 h culture was allowed to ensure the adherence of cells. By supplying SIN-1 (0.5 mM) and PDGF-BB (100 ng/mL) contained medium to the source reagent channel, a stable linear-gradient of conditioning molecules is formed across the microfluidic device. HBVPs were then imaged using real-time video microscopy equipped with a 5 × objective for 12 h (Zeiss Cell discoverer 7). For each condition, three independent trials were performed with ~30 migratory cell tracks recorded per trial. ImageJ software was used to track cell centroid movement of all cells present in the field throughout the time course. The migration tracks of each cell in each group were plotted after normalizing the start point to x0 and y0. Chemotactic index of a cell was defined to be the ratio of the net distance a cell migrates in the direction of the gradient to the total length of the cell track.

### Transwell chamber assays

HBVPs (2 × 10^5^ cells) suspended in 100 μl of DMEM were loaded into the upper well of the Transwell chamber (8 μm pore size, Corning) that was pre-coated with Matrigel (BD Biosciences) at 37 °C. The lower well was filled with 600 μl of DMEM containing 100 ng/mL Wnt7a or 100 ng/mL PDGF-BB as a chemoattractant. After incubation for 12 h, the membrane was fixed with 4% paraformaldehyde (PFA, Sigma-Aldrich, P6148). Nonmigrating cells on the top of the membrane were scraped off with cotton swabs, and migrating cells on the lower face of the membrane were stained with DAPI and cells were recorded and counted in ten areas under 40× magnification using a microscope (Zeiss 800).

### Oxygen and glucose deprivation (OGD) exposure

The oxygen and glucose deprivation (OGD) model was used to mimic the ischemia-like condition in live cells 32. Briefly, the cell culture medium was replaced by glucose-free HBSS (Hank's balanced salt solution), and cells were then placed in a hypoxia chamber (Billups-Rothenberg) containing a gas mixture of 95% N_2_ and 5% CO_2_. Cells were exposed to OGD for 1 h to induce injury. Cells without OGD treatment served as controls.

### Immunocytochemistry

Briefly, cells were fixed in 4% PFA as previously reported 31. The cells were incubated with 0.1% Triton X-100 in PBS for 10 min at RT and for another hour in PBS containing 3% bovine serum albumin (BSA). The cells were incubated with primary antibodies, followed by labeling with corresponding fluorescent secondary antibodies. After staining with DAPI, cells were then rinsed with PBS and mounted onto glass slides with Vectashield mounting medium (Vector Laboratories). Images were captured using a NikonA1R confocal microscope.

The following primary antibodies were used: rabbit anti-GPCR GPR124 (GPR124, 1:300, Abcam, ab67820), rabbit anti-GPR124 (TEM5, 1:500, Invitrogen, PA5-20442), mouse anti-Vinculin (1:300, Abcam, ab18058), mouse anti-Paxillin (1:300, Thermofisher, MA5-13356), Alexa Fluor 647 Phalloidin (1:40, Invitrogen, A22287), Alexa Fluor 555 Phalloidin (1:40, Invitrogen, A34055), mouse anti-Myc-tag (1:2,000, Medical & biological laboratories, M192-3), goat anti-PDGFR-β (1:300, R&D systems, AF1042), rabbit anti-Partitioning-defective 3 (PAR-3, 1:500, Millipore, 07-330).

Isolated focal adhesions were stained with the following antibodies: rabbit anti- GPR124 (1:300, Abcam, ab67820), mouse anti-Vinculin (1:300, Abcam, ab18058), mouse anti-Paxillin (1:300, Thermofisher, MA5-13356).

### Immunohistochemistry

At 72 h after ME, mice were anesthetized and transcardially perfused with PBS, followed by perfusion with cold 4% PFA (w/v) in PBS. Whole brains were immediately removed and fixed overnight at 4 ℃. The specimens were then cryoprotected with 30% sucrose in PBS and sectioned into the 45-μm-thick sample on a freezing microtome 31. The sections were incubated with 0.1% Triton X-100 in PBS for 15 min at RT and for another hour in 3% BSA in PBS. The sections were incubated with primary antibodies, followed by staining with corresponding secondary antibodies. After staining with DAPI, sections were then washed with PBS and mounted onto glass slides with Vectashield mounting medium (Vector Laboratories). Images were acquired using a NikonA1R confocal microscope.

The following primary antibodies were used: rabbit anti-GPCR GPR124 (GPR124, 1:300, Abcam, ab67820), goat anti-CD13 (1:200, R&D systems, AF2335), Fluorescein lycopersicon esculentum (tomato) lectin (1:200, Vector Laboratories, FL-1171), Rabbit anti-Nitrotyrosine (1:1,000, Millipore, 06-284), rabbit anti-Ki67 (1:200, Abcam, ab66155).

### Cell Counting Kit-8 (CCK-8) Assay

Cell viability was measured by CCK-8 assay (Dojindo, CK04) 32. In brief, the normal HBVPs and HEK 293 cells, GPR124-knockout (GPR124-KO) subtypes were seeded into 96-well plates at a density of 2,000 cells/well and cultured until cells filled with the plates. Subsequently, 10 μl of CCK-8 solution was added to each well of the plate and incubated at 37 °C for 2-3 h. Optical adsorption at 450 nm was measured using a microplate reader (Tecan), which showed a positive correlation with the cell viability.

### Stochastic optical reconstruction microscopy (STORM) image acquisition

The super-resolution STORM method was described previously 33. Cells were fixed with 3% PFA (w/v) and 0.1% Isoamyl alcohol for 10 min. The cells were incubated at RT with freshly prepared 0.1% Sodium borohydride in PBS for 7 min under gentle shaking. Cells were then washed three times (5 min at RT each) with PBS followed by blocking with 3% BSA and 0.2% Triton X-100 in PBS for another hour. Cells were incubated with diluted primary antibodies in blocking solution for 1 h at RT. After staining with primary antibodies, the cells were incubated with suitable fluorescent secondary antibodies for 40 min at RT. The secondary antibodies were labeled with an activator-reporter dye (Alexa Fluor 647) pair which were dissolved in DMSO. Afterward, they were washed for three times (10 min each) with PBS containing 0.2% BSA and 0.05% Triton X-100. Next, cells were fixed with 3% PFA (w/v) and 0.1% Isoamyl alcohol for 10 min. After three washes with PBS, cells were then immersed in 200 µl of buffer specialized for STORM imaging. The buffer was freshly prepared before data acquisition, which contained 7 µl of oxygen-scavenging GLOX buffer (14 mg of glucose oxidase, 50 µl of 17 mg/mL catalase in 200 µl of 10 mM Tris, 50 mM NaCl, pH 8.0), 35 µl of MEA buffer (1 M) and 7 µl of β-Mercaptoethanol. All images were acquired on a Nikon N-STORM super-resolution system (Nikon Instruments Inc.) with a Nikon Eclipse Ti inverted microscope. For Z-stack imaging of the cytoskeleton, the images were recorded, processed, and analyzed by using Imaris software (Bitplane, Switzerland).

### Pull-down assay for Cdc42

The assay was performed using Active Cdc42 Pull-Down and Detection Kit (Invitrogen, 16119) according to the manufacturer's protocol. Briefly, cells were chilled on ice and lysed in ice-cold buffer (25 mM Tris-HCl, 150 mM NaCl, 5 mM MgCl_2_, 1% NP-40, 5% glycerol, pH 7.2,) containing protease inhibitor. Lysates were centrifuged at 16,000× g at 4 ℃ for 15 min and the supernatant was collected. An aliquot of each supernatant collected was denatured in SDS-PAGE lysis buffer to measure the total Cdc42 content by Western blotting. For pull-down assays, supernatants containing 500µg of total proteins were incubated with 20 µg of GST-Pak1-PBD protein linked to glutathione resin for 1 h at 4 °C. The resin was washed three times in lysis buffer. The samples were eluted and electrophoresed on 12% SDS-PAGE.

### Statistical analysis

Statistical analysis was performed using the GraphPad Prism software package. All statistical analyses were conducted based on results of biological replicates; the group size (n) is indicated in the figure legends and supplementary table. The results are expressed as the mean ± standard error of the mean (s.e.m.). The significance (P < 0.05) of differences between the two groups were calculated using unpaired Student's t-tests (two-tailed) or one-way ANOVA with Dunnett's multiple comparisons test. The sample size, statistical tests used, and *P* value for each experiment are presented in the table.

## Results

### Microsphere embolism increases pericyte GPR124 expression

**Figure 1:**
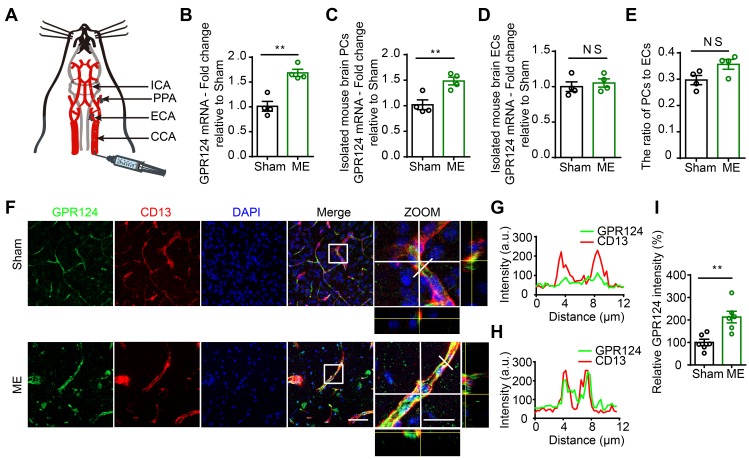
** Changes in GPR124 expression after ME induction in the brain. (A)** Schematic representation of the microsphere embolism (ME) model.** (B-D)** Real-time PCR analysis of GPR124 mRNA expression in the cortical areas of the ipsilateral hemisphere (**B**), isolated primary mouse pericytes (**C**) and endothelial cells (**D**) from Sham and ME mice. The cortical areas of the ipsilateral hemisphere were dissected from mice at 72 h after injection of microspheres. Sham: n = 4; ME: n = 4.** (E)** Real-time PCR analysis of CD13 and CD31 mRNA expression in the cortical areas of Sham and ME mice, indicating the ratio of pericytes to endothelial cells. Sham: n = 4; ME: n = 4. (**F**) Maximum intensity projections of confocal images from brain sections stained for GPR124 (green), CD13 (red) and DAPI (blue, nuclei) at 72 h following ME. The images on the right are magnifications of the boxed regions in the images directly to their left. Scale bar: 50 μm, magnified images, 20 μm. (**G, H**) Plots of the fluorescence intensity in arbitrary units (AU) along the solid lines indicated in the high-magnification images in **F** for Sham (**G**) and ME (**H**), respectively. (**I**) Signal intensities of GPR124 staining quantified from **F** (dot plot, Sham: n = 6; ME: n = 6). *P < 0.05, and **P < 0.01 vs. Sham. The data are presented as the mean ± s.e.m. Statistical significance was determined by unpaired Student's t-test. NS, not statistically significant.

The microsphere embolism (ME) model is a widely used animal model of human mini-stroke (Figure 1A) 2,34. As shown in Figure 1B, quantitative Real-Time PCR (qRT-PCR) revealed a ~1.6-fold increase in GPR124 transcript in the cortical areas in ME mice compared to sham-operated mice. To identify which cell types were subjected to the increase in GPR124 signaling, we evaluated the mRNA level of GPR124 in isolated mouse brain pericytes (PCs) and endothelial cells (ECs) from Sham or ME mice. The data showed that GPR124 was selectively upregulated in PCs comparing to insignificant variation as observed in ECs (Figure 1C-D). In addition, qRT-PCR analysis of the expression of CD13 (PCs marker) and CD31 (ECs marker) revealed that the relative ratio of PCs to ECs did not change in ME and Sham mice (Figure 1E).

Next, we used dual-immunostaining for cell-specific markers to examine the cellular localization of GPR124 at 72 h after microsphere injection. There was a significant increase in nitrotyrosine (Figure S1A-B) and ki67 (Figure S1C-D) fluorescence intensity in response to microvessel injury, indicating enhanced nitrosative stress and cell proliferation in pericytes. Moreover, we found that the expression of GPR124 was significantly increased in CD13-positive pericytes surrounding occluded microvessels (Figure 1F-I). Overall, these data suggest that robust GPR124 expression in pericytes is predominantly associated with vessel remodeling after microcapillary embolism.

### Localization of GPR124 in human brain vascular pericytes

**Figure 2:**
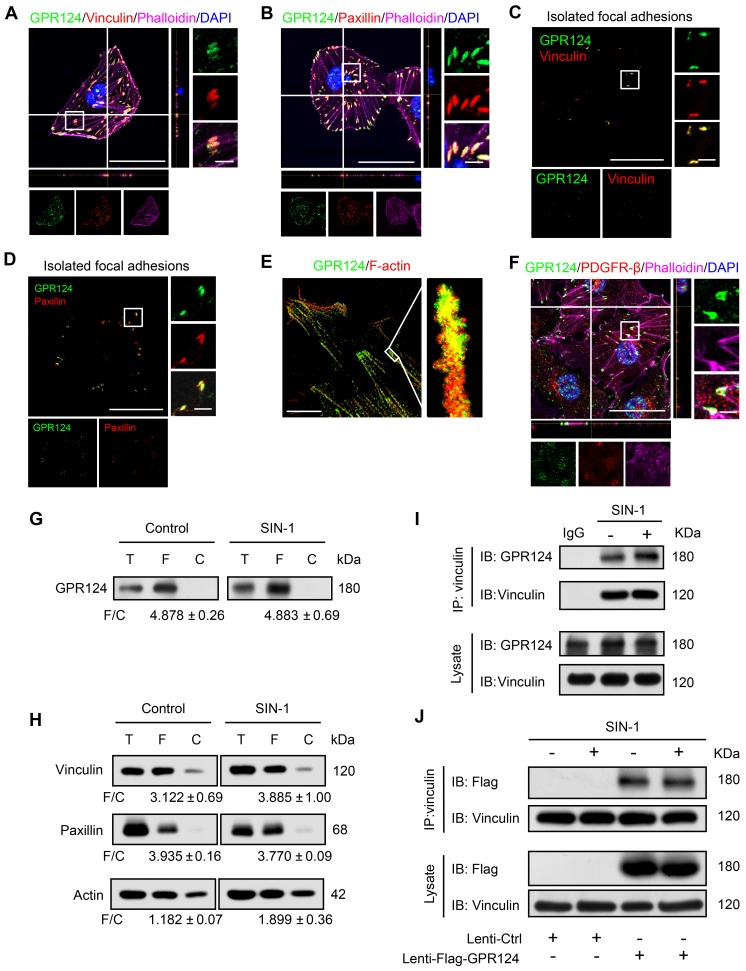
** GPR124 expression in focal adhesions in HBVPs.** (**A, B**) Maximum intensity projections of confocal images from HBVPs stained for GPR124 (green), Vinculin (red, **A**), Paxillin (red, **B**), Phalloidin (magenta) and DAPI (blue, nuclei). The images on the right panel are magnifications of the boxed regions in the images. Scale bar: 40 μm (Left), magnified images, 5 μm. Representative stainings from three independent experiments are shown. (**C, D**) Isolated focal adhesions include the same proteins as focal adhesions found in intact HBVPs. Confocal fluorescence microscopy images of immunostained isolated focal adhesions demonstrating the presence of GPR124 (green), Vinculin (red, **C**) and Paxillin (red, **D**). Scale bar: 40 μm (Left), magnified images, 5 μm. (**E**) The localization of GPR124 and F-actin in HBVPs was observed by STORM. Note that there is a close interaction between GPR124 and the F-actin terminals. Scale bar: 10 μm. (**F**) Maximum intensity projections of confocal images from primary mouse brain pericytes stained for GPR124 (green), PDGF-β (red), Phalloidin (magenta) and DAPI (blue, nuclei). The images on the right panel are magnifications of the boxed regions in the images. Scale bar: 40 μm (Left), magnified images, 5 μm. (**G, H**) Western blot comparison of protein concentration in total cell lysate (T), isolated focal adhesion fractions (F) and cell body fractions (C). Equal total protein was loaded in each lane. The ratio shown on the lower (F/C) indicates the relative concentration of protein between the isolated focal adhesion and cell body fractions quantified from the intensities of bands from western blots. n =3 in each group. The data are presented as the mean ± s.e.m. Note that these only reflect the relative amount of protein in isolated focal adhesion fractions to cell body fractions. (**I**) Endogenous GPR124 binding to Vinculin in HBVPs incubated with or without SIN-1 (0.5 mM, 12 h). Immunoprecipitated Vinculin was immunoblotted with GPR124 and Vinculin. (**J**) Exogenous GPR124 binding to Vinculin in HBVPs. The HBVPs transfected with Flag-GPR124 and treated in the presence or absence of SIN-1 (0.5 mM, 12 h).

To clarify the role of GPR124 in pericytes in detail, we cultured human brain vascular pericytes (HBVPs) and performed immunofluorescence staining using antibodies against GPR124, focal adhesions specific markers including vinculin and paxillin 35,36, as well as the cytoskeletal marker Phalloidin. Our data demonstrated that GPR124 was specifically expressed in focal adhesion plaques (Figure 2A-B).

Focal adhesions are large protein complexes that maintain the cell polarity and mediate migration through assembly and disassembly of the cytoskeleton 37,38. Experiments with isolated focal adhesions confirmed the colocalization of GPR124 with the focal adhesion proteins vinculin and paxillin (Figure 2C-D). Interestingly, the stochastic optical reconstruction microscopy (STORM) results also showed that GPR124 colocalized with F-actin terminals (Figure 2E). We also observed an enrichment of GPR124 in the focal adhesion in primary cultured pericytes (Figure 2F). Consistently, western blot data also showed GPR124 localized in the isolated focal adhesion complex (Figure 2G). We compared the level of focal adhesion proteins (paxillin, vinculin, actin) in total cell lysate (T), isolated focal adhesion (F) and cell body fractions (C) by western blot (Figure 2H), which confirmed that the isolation method concentrated focal adhesion components in the focal adhesion fraction.

During ischemia pathological processes, ONOO^-^, a highly reactive nitrogen species, is generated from the reaction between nitric oxide (NO) and superoxide (O_2_^-^) 39. To evaluate the interaction between GPR124 and focal adhesions proteins and whether ONOO^-^ affects the interaction, coimmunoprecipitation (co-IP) experiments were performed. We verified that vinculin interacted with GPR124 and this interaction was not affected under nitrosative stress (Figure 2I). The occurrence of the interaction was also further confirmed by the overexpressing GPR124 3×flag constructs in HBVPs (Figure 2J). Taken together, these results suggest that GPR124 may spatiotemporally link the focal adhesions and the actin cytoskeleton through its interaction with focal adhesions proteins. GPR124 may serve as an important regulatory factor for cell adhesion and migration.

### Redistribution of GPR124 in polarized pericytes upon stress

**Figure 3:**
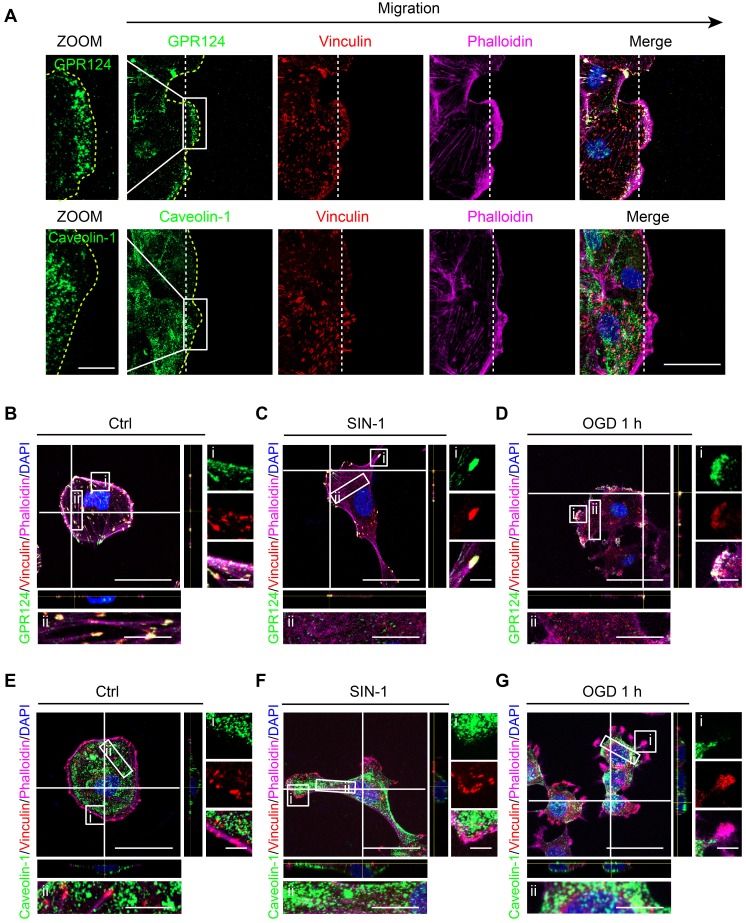
** GPR124 distribution is altered following ischemia-like injury.** (**A**) Representative images showing GPR124 (green, upper panel), Caveolin-1 (green, lower panel), Vinculin (red), Phalloidin (magenta) and DAPI (blue, nuclei) in the leading edge of indicated HBVPs at 4 h after scratch. The dashed line indicates the adjacent front of reorienting cells. Note that the expression of GPR124 is significantly increased in the pseudopodia of cells in the direction of migration. Scale bar: 20 μm, magnified images, 10 μm. (**B-G**) Fluorescence redistribution of GPR124 (**B, C, D**) and Caveolin-1 (**E, F, G**) in HBVPs treated with or without SIN-1 (0.5 mM, 12 h) and OGD (1 h). The **i** and **ii** shows higher-magnification images of the boxed regions in the image. Scale bar: 40 μm, 5 μm (**i**) and 10 μm (**ii**).

During wound healing scratch, cells migrate through polarization based on extracellular cues that guide directional movement 37. Here, we used caveolin-1 as a control, which was the most abundant protein on the cell membrane 40. We found that scratch stimulation-induced GPR124 redistribution to the leading edges of the leader pericytes in the migrating monolayer while having no effect on caveolin-1 (Figure 3A).

Cerebral ischemia is known to cause hypoxia, glucose deprivation, and reactive oxygen or nitrogen species 41. Upon nitrosative stress, cells form distinct front and rear areas characterized by leading edges with membrane protrusion and retracting tails, respectively; this process is known to be a prerequisite for polarized cell to move 42. We next examined whether reactive nitrogen species and glucose deprivation could have an impact on the localization of GPR124. In this case, HBVPs were incubated with SIN-1 (0.5 mM) for 12 h. Following the treatment, GPR124 appeared to be restricted to the leading edges of cell protrusions and colocalized with vinculin (Figure 3B-C). Similar results were obtained following OGD (Figure 3D). In contrast, diffuse distribution of caveolin-1 was observed in the cytoplasm but not in focal adhesions (Figure 3E-G).

Together, these findings indicate that GPR124 at the leading edge may participate in polarized cytoskeletal rearrangements to facilitate directional migration.

### GPR124-dependent signaling is essential for directional migration of pericytes

**Figure 4:**
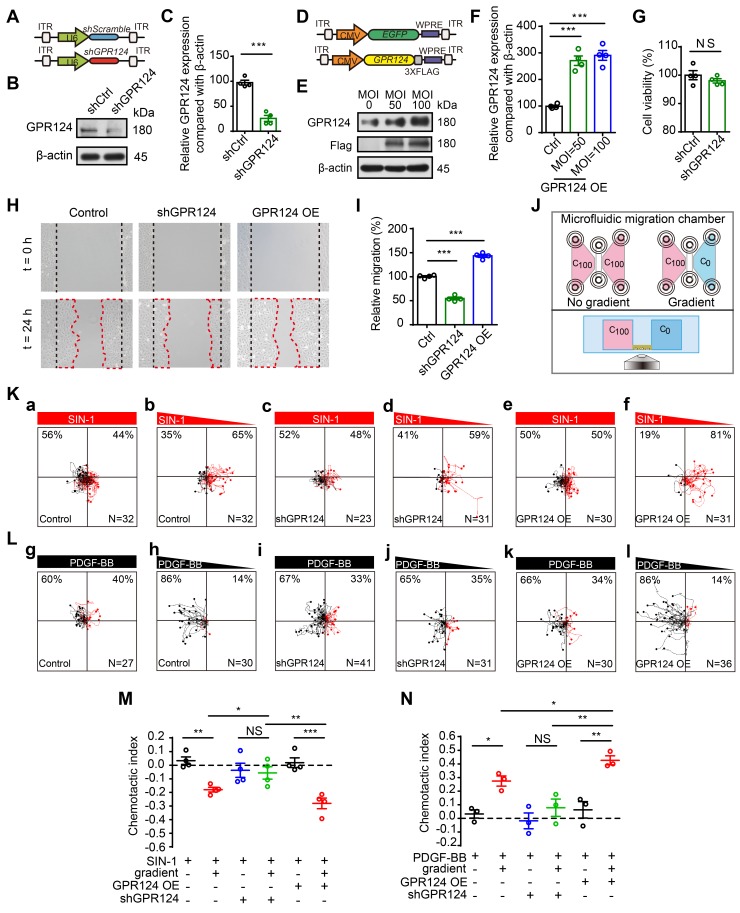
** GPR124-dependent signaling controls pericyte migration.** (**A**) Diagram of the knockdown system. (**B**) Immunoblot analysis of GPR124 protein level in control HBVPs and in HBVPs transduced with a lentivirus expressing an shRNA to silence endogenous GPR124. (**C**) Quantitative analyses of the results as shown in (**B**) and presented in the bar graph as the densitometry ratio of the control from four independent experiments. (**D**) Diagram of the overexpression system. (**E**) Immunoblot analysis of GPR124 proteins in HBVPs transfected with either control or gene construct encoding GPR124 (FLAG-GPR124, MOI = 50 and 100) to confirm overexpression of GPR124. (**F**) Quantitative analyses of the results as shown in (**E**) and presented in the bar graph as the densitometry ratio of the control from four independent experiments. (**G**) Cell viability was measured by the CCK8 assay in Ctrl-HBVPs and shGPR124-HBVP (n = 4). **(H)** Representative images of control HBVPs (control), shGPR124 HBVPs (shGPR124) and GPR124-overexpressing HBVPs (GPR124 OE) at the beginning (t = 0 h) and end (t = 24 h) of the wound healing assay. The dashed lines (black) indicate the edges of the wound immediately after scratch. The dashed lines (red) outline the area of cell migration. **(I)** Quantification of relative migration in a wound healing assay after 24 h (normalized to control values; n = 4 in each group).** (J)** Schematic representation of the microfluidic migration chamber used for observing the chemotactic response of cells exposed to chemical gradients. (**K**) Role of GPR124 on the migratory response of HBVPs to SIN-1. Control and GPR124 OE HBVPs exhibited migration backward gradients of SIN-1 (0.5 mM, **b,f**), but migrated randomly to equivalent concentration (no gradient,** a,e**). No directed migration was observed in shGPR124 HBVPs (**c-d**). Tracks of individual cells are shown. (**L**) Role of GPR124 on the migratory response of HBVPs to PDGF-BB. Control and GPR124 OE HBVPs exhibited migration toward gradients of PDGF-BB (100ng/mL, **h,l**), but migrated randomly to equivalent concentration (no gradient,** g,k**). No directed migration was observed in shGPR124 HBVPs (**i-j**). Tracks of individual cells are shown. (**M**, **N**) Dot plot of chemotactic indices of HBVP migration. *P < 0.05, **P < 0.01 and ***P < 0.001 vs. control. The data are presented as the means ± s.e.m. Statistical significance was determined by unpaired Student's t-test (for **C, F, G, I**) and one-way ANOVA with Dunnett's multiple comparisons test (for **M, N**).

The above described redistribution of GPR124 in polarized pericytes suggests that GPR124 signaling is essential for stress-induced pericytes migration. To verify this possibility, shRNA-GPR124 (shGPR124) and GPR124 overexpressing (GPR124 OE) lentivirus were constructed to detect effects on cell migration. Endogenous GPR124 expression was suppressed using lentivirus-mediated RNA interference (RNAi) (Figure 4A-C and Figure S2A-D). Immunoblot analysis verified that GPR124 protein levels were significantly upregulated in HBVPs by the transfection of GPR124 OE lentivirus (Figure 4D-F). In addition, we found that the cell viability of shGPR124-expressing HBVPs was comparable to shSramble cells using CCK-8 assay (Figure 4G). Consistent with our hypothesis, GPR124-knockdown significantly decreased pericyte motility, whereas the GPR124 overexpression significantly increased it (Figure 4H-I). In addition, GPR124 was found to colocalize with Partitioning-defective 3 (PAR-3) (Figure S2E), a protein known to be essential for the growth of polarized cells 43.

We further examined the effect of GPR124 overexpression and GPR124 knockdown on HBVPs using a microfluidic migration chamber with extremely stable, shear-minimized linear chemoattractive or chemorepulsive gradients (Figure 4J). Previous studies have shown that attractive and repulsive cell guidance was important in the pathological process of diseases 44-47. Here, HBVPs migrated away from SIN-1 were observed, indicating that SIN-1 was a repellant guidance cue of HBVPs (Figure 4K, a-b). Attracting by PDGF-BB, pericytes moved toward the source of PDGF-BB (Figure 4L, g-h). However, the directed migration of HBVPs was not observed upon shGPR124 knockdown (Figure 4K, c-d and 4L, i-j). GPR124 OE HBVPs exhibited directed migration in microfluidic chambers backward gradients of conditioned medium containing SIN-1 (Figure 4K, f and M), but cells migrated randomly when presented with equivalent SIN-1 conditioned medium (no gradient) (Figure 4K, e). In addition, GPR124 OE HBVPs was more sensitive to the PDGF-BB (100 ng/mL) gradient and exhibited directed migration in microfluidic chambers toward gradients of PDGF-BB-conditioned medium (Figure 4L, k-l and N). Taken together, our findings demonstrate that GPR124 plays an important role in pericytes migration, which exerts a significant promigratory effect on pericytes.

### GPR124 regulates filopodia formation and cell polarity in pericytes

The initial steps in classic models of cell migration include cell polarization and extension of cell leading edges in protrusions 48. Considering that GPR124 participates in polarized cytoskeletal rearrangements and has a significant promigratory effect, we hypothesized that GPR124 might participate in the initial step of cell migration. To determine if GPR124 is required for filopodia formation and cell polarization induced by nitrosative stress, GPR124 loss-of-function studies were performed in HBVPs and human embryonic kidney 293 (HEK293) cells using CRISPR-Cas9-mediated gene deletion (Figure S3A). Cas9 and sgRNA complexes induced GPR124 DNA double-strand breaks (DSBs) that could be repaired through the NHEJ pathway, causing indels 49. As shown, inappropriate peaks could be found in the sequences of PCR products amplified from the GPR124 gene in HBVP and HEK293 cell clones edited by CRISPR (Figure S3B-C). Both western blot and immunostaining data showed that the expression of GPR124 was effectively reduced in GPR124-knockout (GPR124-KO) HBVPs and HEK293 cells (Figure S3D-E and G-J). We also found that the proliferative ability was comparable in GPR124-KO HBVPs to that of control cells (Figure S3F). In addition, PDGF-BB enhanced the directional migratory ability of HBVPs as reflected by the transwell assay and this migratory response to PDGF-BB declined substantially in GPR124-KO HBVPs (Figure S4A-B).

Accordingly, co-staining with phalloidin and VASP was used to determine the filopodia formation in control and GPR124-KO HBVPs. VASP is a specific filopodia marker, which is dominantly concentrated at the tips of filopodia, the leading edge of lamellipodia, as well as in focal adhesions 50.

**Figure 5:**
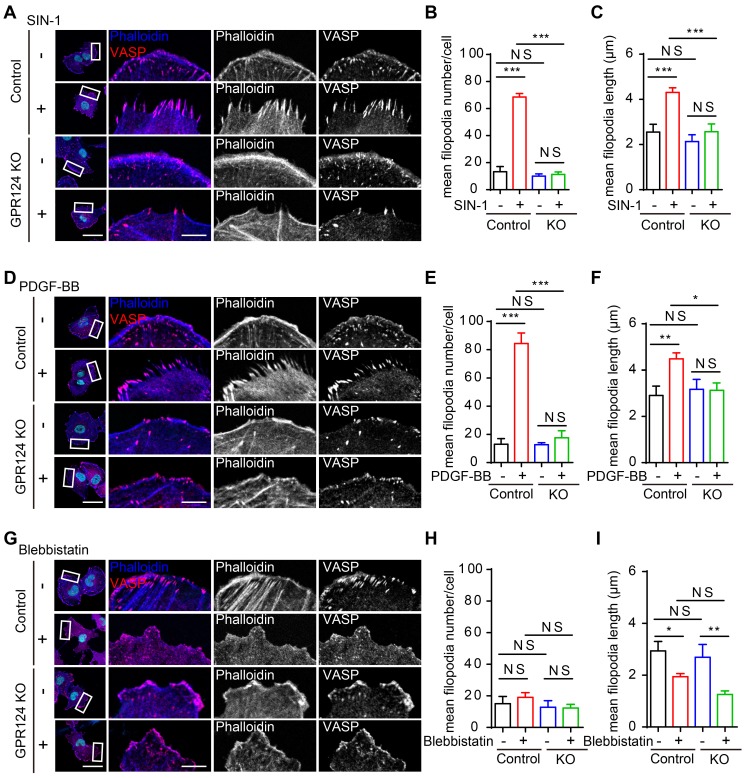
** GPR124 regulates filopodia formation in HBVPs in vitro.** (**A-C**) Representative images and quantitative analysis of HBVPs lacking GPR124 treated with SIN-1 (0.5 mM). Cells were stained with Phalloidin (blue), VASP (red) and DAPI (cyan, nuclei). Right panels, magnification of boxed regions. Scale bar: 40 μm; magnified images, 10 μm. Quantification of VASP-labeled filopodia for mean filopodia number per cell (**B**, n = 9, 18, 9, 9) and mean length of filopodia (**C**, n = 11, 16, 11, 10) from experiments shown in **A**. (**D-F**) Representative images and quantitative analysis of HBVPs lacking GPR124 treated with PDGF-BB (100 ng/mL). Cells were stained with Phalloidin (blue), VASP (red) and DAPI (cyan, nuclei). Right panels, magnification of boxed regions. Scale bar: 40 μm; magnified images, 10 μm. Quantification of VASP-labeled filopodia for mean filopodia number per cell (**E**, n = 12, 12, 12, 12) and mean length of filopodia (**F**, n = 12, 30, 12, 11) from experiments shown in **D**. (**G-I**) Representative images and quantitative analysis of HBVPs lacking GPR124 treated with blebbistatin (20 μM). Cells were stained with Phalloidin (blue), VASP (red) and DAPI (cyan, nuclei). Right panels, magnification of boxed regions. Scale bar: 40 μm; magnified images, 10 μm. Quantification of VASP-labeled filopodia for mean filopodia number per cell (**H**, n = 18, 15, 12, 9) and mean length of filopodia (**I**, n = 12, 17, 11, 19) from experiments shown in **G**. *P < 0.05, **P < 0.01, and ***P < 0.001 vs. control. The data are presented as the means ± s.e.m. Statistical significance was determined by one-way ANOVA with Dunnett's multiple comparisons test. NS, not statistically significant.

Quantitative analysis showed that the mean filopodia number per cell and the mean filopodia length increased markedly after incubation of HBVPs with SIN-1 (0.5 mM, Figure 5A-C) and PDGF-BB (100 ng/mL, Figure 5D-F), but this increase in filopodia was abolished upon genetic GPR124 deletion. In addition, our data suggested that there was no difference in the number of filopodia while the length of filopodia was significantly decreased upon treatment of control HBVPs with Blebbistatin (20 μM). The length of filopodia was unaltered in GPR124-KO HBVPs compared with control cells received Blebbistatin (Figure 5G-I). Indeed, alongside the alterations in filopodia, GPR124 deletion also significantly decreased PAR-3 positive signals at focal adherent junctions (Figure S4C-D).

Collectively, these findings suggest that genetic GPR124 deletion from pericytes inhibits filopodia formation and cell polarization at the onset of nitrosative stress.

### Time-lapse imaging reveals that GPR124 contributes to filopodia formation upon nitrosative stress

The role of actin and microtubule networks in cell migration of single cells has been well characterized 51,52. Previous studies have shown that GPR124 influences the spread of filopodia during angiogenesis 11. Filopodia are actin-based, finger-like protrusions that can support slow, continuous migration 53. Consistent with the results of a previous study 17, HEK293 cells were found to express endogenous GPR124 herein (Figure S3I).

**Figure 6:**
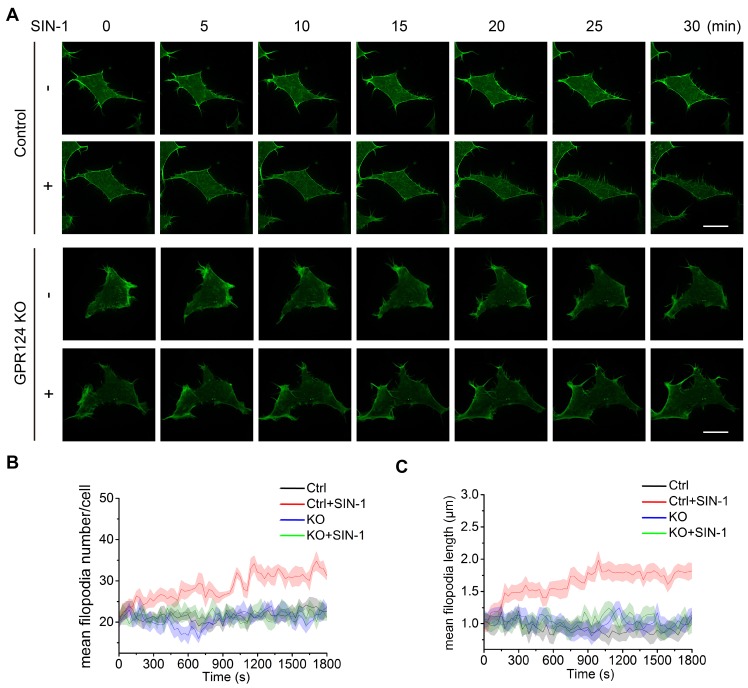
** GPR124 regulates filopodia formation in HEK293 cells upon nitrosative stress.** (**A**) Time-lapse series of single confocal plane images taken of living normal HEK293 cells and GPR124-KO HEK293 cells. The cells were seeded on 35-mm glass-bottom dishes overnight and then stimulated with or without SIN-1 (0.5 mM) for 30 min. Scale bar: 20 μm. (**B, C**) Quantification of dynamic changes in the numbers of filopodia (**B**) and the lengths of filopodia (**C**) after SIN-1 (0.5 mM) treatment (n=30).

To focus on the primary role of GPR124 in filopodia formation upon nitrosative stress, we investigated the differences in filopodia formation between wild-type and GPR124-KO HEK293 cells by labeling filamentous actin with Lifeact-EGFP (Figure 6A). The number of filopodia and the length of filopodia steadily increased in HEK293 cells upon SIN-1 stimulation (0.5 mM). In contrast, we did not observe changes in filopodia number or filopodia length in GPR124-deficient HEK293 cells (Figure 6B-C), indicating that GPR124 was required and necessary for the filopodia formation. Therefore, our real-time imaging system allows us to monitor the dynamic changes at high spatial and temporal resolutions. Together, these results suggest a requirement for GPR124 in the regulation of actin cytoskeleton organization and filopodia formation during cell stress.

### The Horm domain of GPR124 is required for filopodia formation

To explore the functional domains required for GPR124-mediated filopodia formation, we constructed a series of deletion mutants for each of the domains and investigated the localization of each mutant in transfected cells (Figure S5A). Western blot analysis was performed to confirm expression of the different constructs in GPR124-KO HBVPs (Figure S5B).

**Figure 7:**
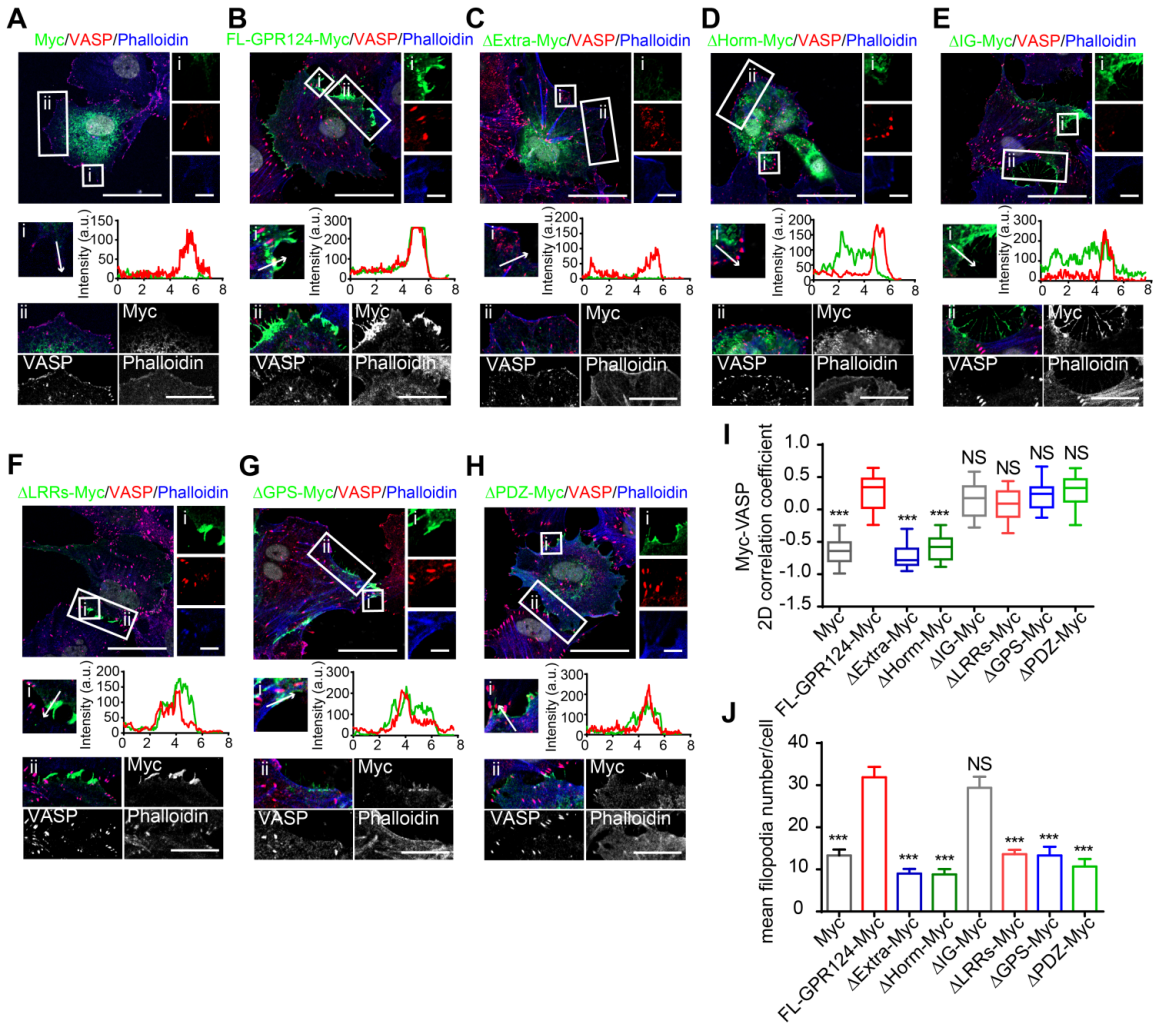
** Essential domain of GPR124 required for SIN-1-induced pericyte migration. (A-H)** Confocal images of Myc (green), VASP (red), Phalloidin (blue) and DAPI (gray) in cultured HBVPs. GPR124-KO HBVPs transfected with FL-GPR124-Myc or Myc-tagged GPR124 truncation mutants as indicated. The **i** and **ii** shows higher-magnification images of the boxed regions in the image. Plots of the fluorescence intensity in arbitrary units (AU) along the solid lines indicated in the high-magnification images in **i**. Scale bar: 40 μm, 5 μm (**i**) and 10 μm (**ii**). (**I**) 2D correlation coefficient between Myc and VASP signals (box plot; n = 26). (**J**) Quantification of the mean filopodia number per cell and mean length of filopodia (n = 20). ***P < 0.001 vs. FL-GPR124-Myc. The data are presented as the mean ± s.e.m. Statistical significance was determined by unpaired Student's t-test. NS, not statistically significant. SP, signal peptide; LRRs, leucine-rich regions; Ig, immunoglobulin (Ig)-like domain; Horm, hormone-binding domain; GPS, GPCR proteolytic site; 7TM, seven-pass transmembrane domains; PDZ, PDZ-binding motif.

The distribution of full-length Myc-tagged GPR124 (referred to as FL-GPR124-Myc) and its mutants were examined in GPR124-KO HBVPs after treatment with SIN-1 (0.5 mM) for 12 h. Immunostaining was performed with an anti-Myc antibody (representing for GPR124 protein), anti-VASP antibody and phalloidin (Figure 7A-H). We observed that robust GPR124 expression at the edge of cell membrane (Figure 7B). All GPR124 mutants exhibited a similar pattern of expression except for constructs with the deletion of either the extracellular domain (Figure 7C) or the Horm domain (Figure 7D) (referred to as ΔExtra-Myc or ΔHorm-Myc, respectively). Deletion of the two domains resulted in diffuse expression of GRP124. The 2D correlation coefficient between the Myc-tagged proteins and VASP signals were calculated (Figure 7I), indicating that deletion of Horm was sufficient to attenuate the function of GPR124 in cell polarity.

Additionally, the mean filopodia number per cell was significantly decreased in cells expressing GPR124 mutant-Myc except for that with its Ig domain depleted (referred to as ΔIg-Myc) (Figure 7J), indicating that the Ig domain was unnecessary for supporting the GPR124-mediated filopodia formation. We also transfected full-length Myc-tagged GPR124 and its mutants into GPR124-KO HEK293 cells and treated the cells with SIN-1 (0.5 mM) for 4 h. As expected, the results were similar to that of HBVPs (Figure S5C-E). The functions of different constructs in cell migration were further confirmed by using wound healing assay (Figure S6, A-B for HEK293 cells and C-D for HBVPs).

Taken together, these data suggest that deletion of the Horm domain largely eliminate the function of GPR124 in pericytes, whereas the deletion of LRRs, GPS or PDZ-binding motif have no effect in terms of GPR124 distribution but influence the filopodia formation and pericyte migration.

### GPR124 mediated Cdc42-dependent directional migration

**Figure 8:**
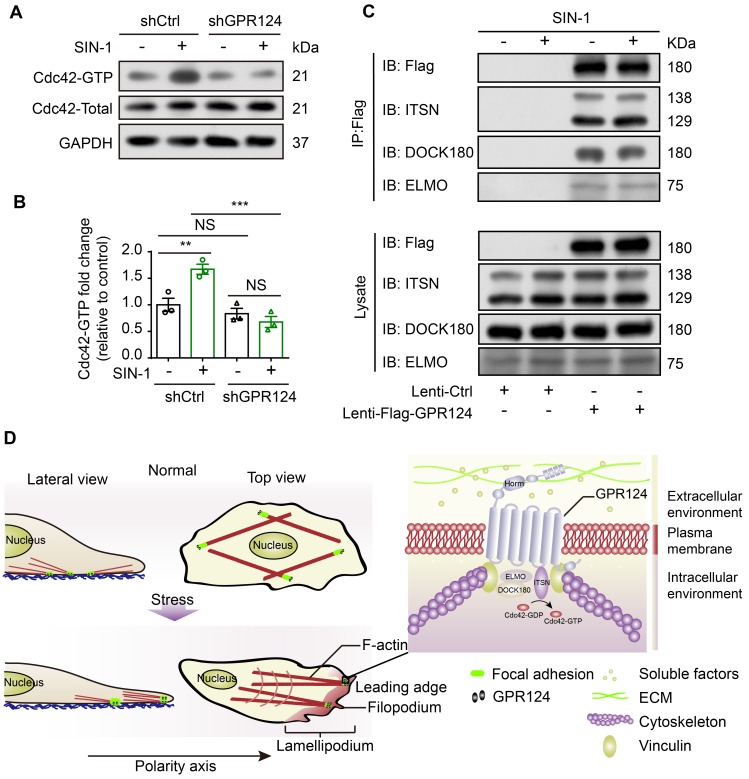
** Role of GPR124 in pericyte polarization and migration.** (**A**) Immunoblot analysis of Cdc42-GTP (upper panel) and Cdc42-Total (Lower panel) in shCtrl-HBVPs and shGPR124-HBVPs cells treated with or without SIN-1. GAPDH used for normalization. (**B**) Densitometric analysis of active Cdc42 levels. For pull-down quantification, values were calculated as the ratio of Cdc42-GTP to total Cdc42 form and normalized to the values obtained with the control (Ctrl). Data are expressed as mean ± s.e.m. from four independent experiments (n = 3). (**C**) Exogenous GPR124 binding to ITSN, DOCK180 and ELMO in HBVPs transfected with Flag-GPR124 incubated with or without SIN-1 (0.5 mM) for 12 h. Flag was IP followed by IB for Flag, ITSN, DOCK180 and ELMO. (**D**) Schematic representation of the distribution of GPR124 in HBVPs under normal or nitrosative stress conditions. GPR124 localizes to focal adhesions and affects cell polarization by regulating polarized cytoskeletal rearrangements under conditions of cerebrovascular injury. A high-magnification view of the focal adhesion is shown. **P < 0.01 vs. control. Statistical significance was determined by one-way ANOVA with Dunnett's multiple comparisons test.

Spatiotemporal rearrangement of the actin cytoskeleton triggered by the small Rho GTPases of the Ras superfamily dictates actin-driven cellular events. Especially, Cdc42 GTPase cooperates with actin assembly to induce the filopodia formation 54. Pulldown assays were performed to analyze the activation of Cdc42. Higher amounts of GTP-bound Cdc42 were detected after SIN-1 stimulation, whereas it was attenuated following GPR124 knockout (Figure 8A-B). Therefore, Cdc42 GTPase might contribute to the decreased migration phenotype observed in GPR124-KO HBVPs.

Furthermore, we examined the possible binding interactions of GPR124 with ELMO, DOCK and Intersectin (ITSN) in HBVPs using co-IP experiments. We used anti-Flag antibodies to immunoprecipitate Flag-GPR124 from lysates of GPR124 overexpressing HBVPs. Figure 8C verified a direct interaction of GPR124 with ELMO, DOCK and ITSN in the HBVPs during nitrosative stress. Thus, GPR124 might induce Cdc42-dependent directional migration of pericytes via its interaction with ELMO, DOCK and ITSN complexes.

## Discussion

Cell adhesion and migration require precise signal transduction through communication between the intracellular signaling pathway and stress environment 55. In this paper, we show that pericytes express functional GPR124 and that, strikingly, GPR124 is localized to focal adhesions and affects cell polarization by regulating the rearrangements of polarized cytoskeletal under cerebrovascular injury condition. A schematic illustrating the distribution of GPR124 in HBVPs under physiological or SIN-1-treated conditions can be found in Figure 8D. These data are consistent with previous findings wherein GPR124 is proved to be available in both ECs and pericytes during mouse embryogenesis and is prominently expressed in the brain and neural tube rather than in non-CNS organs, as confirmed by flow cytometry and immunofluorescence staining 7,9,10.

It is interesting to note that GPR124 colocalized with F-actin and vinculin in focal adhesions, as determined by immunofluorescence staining of endogenous GPR124 in HBVPs. Integrin-based extracellular matrix (ECM) adhesion complexes and cadherin-based cell-cell junction complexes mediate cellular functions involving biochemical and physical interactions between cells and extracellular environment 56,57. Vinculin, which is activated by protein stretching, is a core mechanosensory compartment that is conserved between the two types of complexes 36. The close interaction between GPR124 and cytoskeletal terminals within adhesion plaques suggests that GPR124, as a cell surface membrane receptor, may be one of the key components of cell adhesion complexes. GPR124 may spatiotemporally link the ECM and the actin cytoskeleton through the plasma membrane and act as a molecular scaffold that senses signaling and mechanical forces in addition to receiving external stimuli within the cells.

Interestingly, GPR124 participates in the polarization process of pericytes upon nitrosative stress stimulation. Nitrosative stress induced tyrosine nitration of target proteins during pericyte injury 58. Our study demonstrates that SIN-1 treatment or glucose deprivation in pericytes leads to nitrosative stress and alters GPR124 distribution. Interestingly, we notice that after SIN-1 incubation or starvation-induced cell polarization, GPR124 is largely restricted to leading edges regions of cell membrane protrusions. Along with polarized cell protrusion and cell body retraction, specific adhesions attachment to the ECM has long been considered a necessary step during cell migration 47. Meanwhile, cell surface adhesion receptors interact with the cytoskeleton and transduce signals, control cell morphology, migration and differentiation 59,60. Cell migration during extensive development, homeostasis, and disease processes and has been involved in tumor spread 61. In this study, it was determined that GPR124-dependent regulation of the filopodia formation controlled the directed migration of polarized cells in a two-dimensional matrix microenvironment. In agreement with the results reported here, other studies with a focus on the role of GPR124 in cell migration in different systems revealed that exogenous GPR124 overexpression in mouse and human brain ECs can markedly stimulate cell migration in a Cdc42-dependent manner 7,9,62. Previous studies have shown that the phenotype of global GPR124-KO is indeed more severe than that of endothelium-specific GPR124-KO counterpart 7,12. Therefore, it will be interesting to explore whether pericytes lacking GPR124 can still contribute to the formation of vascular structures and the BBB. The observed severe phenotype is most likely due to the role of GPR124 in cell migration and differentiation. Future studies are needed to uncover the roles of GPR124 in different types of cells in vivo.

Understanding the mechanism by which GPR124 affects the migration of pericytes has great impact on the treatment of the cerebrovascular injury. One major contribution is that the GPR124 signaling indeed drives pericyte migration through modulating the filopodia formation. Cells are first polarized to form a single front based on a single region of actin polymerization. Finger-like membrane protrusions, also known as filopodia, are often found at the leading edges of migrating cells 49. In particular, cytoskeletal rearrangement can be induced by the small GTPase Cdc42, promoting integrin binding with the ECM 63. Using CRISPR/Cas9-engineered cells, we further showed that GPR124 could regulate filopodia formation and cell polarity in HBVPs and HEK293 cells during nitrosative stress. Such an idea has also been supported by in vivo studies with zebrafish GPR124 mutants in which actin-rich structures at the dorsal walls of primordial hindbrain channels where angiogenic tip cells emerged were not observed in GPR124 mutants 11. This study provides the first evidence that GPR124-mediated control over cytoskeletal rearrangement is critical to filopodia formation to promote a polarized migration of pericyte encountering damage factors.

GPR124 has a long N-terminal extracellular domain that is important for protein-protein or adhesion-type interactions. Taking advantage of genetic tools, we directly assessed the functional domains behind the adhesion and migration regulated by GPR124. Our domain mapping analysis suggests that the Horm domain is required for the localization of GPR124 in lamellipodia. Previous studies reveal that the deletion of the LRRs and/or Ig domain abolishes GPR124 binding to the N-terminal domain of Reck, while deletion of the Horm domain approximately halved the strength of canonical signaling 18. The Horm domain contains an RGD (Arg-Gly-Asp) motif. Proteolytic exposure of the cryptic RGD site in GPR124 has been shown to be responsible for its binding to integrin a_v_β_3_. It is postulated that this interaction regulates the adhesion and migration of cells during angiogenesis 64,65. Further investigation is required to uncover the full role Horm domain plays in signal transduction of GPR124 and the communications between ECs and pericytes under physiological and pathological conditions.

## Conclusions

GPR124 is highly expressed in pericytes and located specifically at focal adhesions. Our data demonstrate that, for the first time, GPR124 participates in the filopodia formation, which is essential for supporting pericyte polarization and migration in the context of ischemia-like injury. Pericyte GPR124 may thus be a potential therapeutic target for brain diseases involving neurovascular reconstruction.

## Supplementary Material

Supplementary figures and table.

## Abbreviations

GPR124: G protein-coupled receptor 124
SIN-1: 3-morpholino-sydnonimine
ME: microsphere embolism
ECA: external carotid artery
PPA: pterygopalatine artery
CCA: common carotid artery
BBB: blood-brain barrier
CNS: central nervous system
aGPCR: adhesion-type G protein-coupled receptor
TM: transmembrane
LRRs: leucine-rich repeats
Ig: immunoglobulin
Horm: hormone-binding domain
GPS: GPCR proteolytic site
qRT-PCR: quantitative Real-Time PCR
PCs: pericytes
ECs: endothelial cells
STORM: stochastic optical reconstruction microscopy
OGD: oxygen-glucose deprivation
HBVPs: human brain vascular pericytes
PAR-3: partitioning defective 3 homolog
RNAi: RNA interference
DSB: DNA double-strand breaks
HEK293: human embryonic kidney 293
ECM: extracellular matrix
co-IP: coimmunoprecipitation
