# Supplementary material for: GPR124 facilitates pericyte polarization and migration by regulating the formation of filopodia during ischemic injury
Source: Theranostics. 2019 Aug 14;9(20):5937–55. doi: 10.7150/thno.34168 (PMC6735362; doi:10.7150/thno.34168)
Supplement: Supplementary file 1 — Supplementary figures and table. [file thnov09p5937s1.pdf]

Supplementary Figures

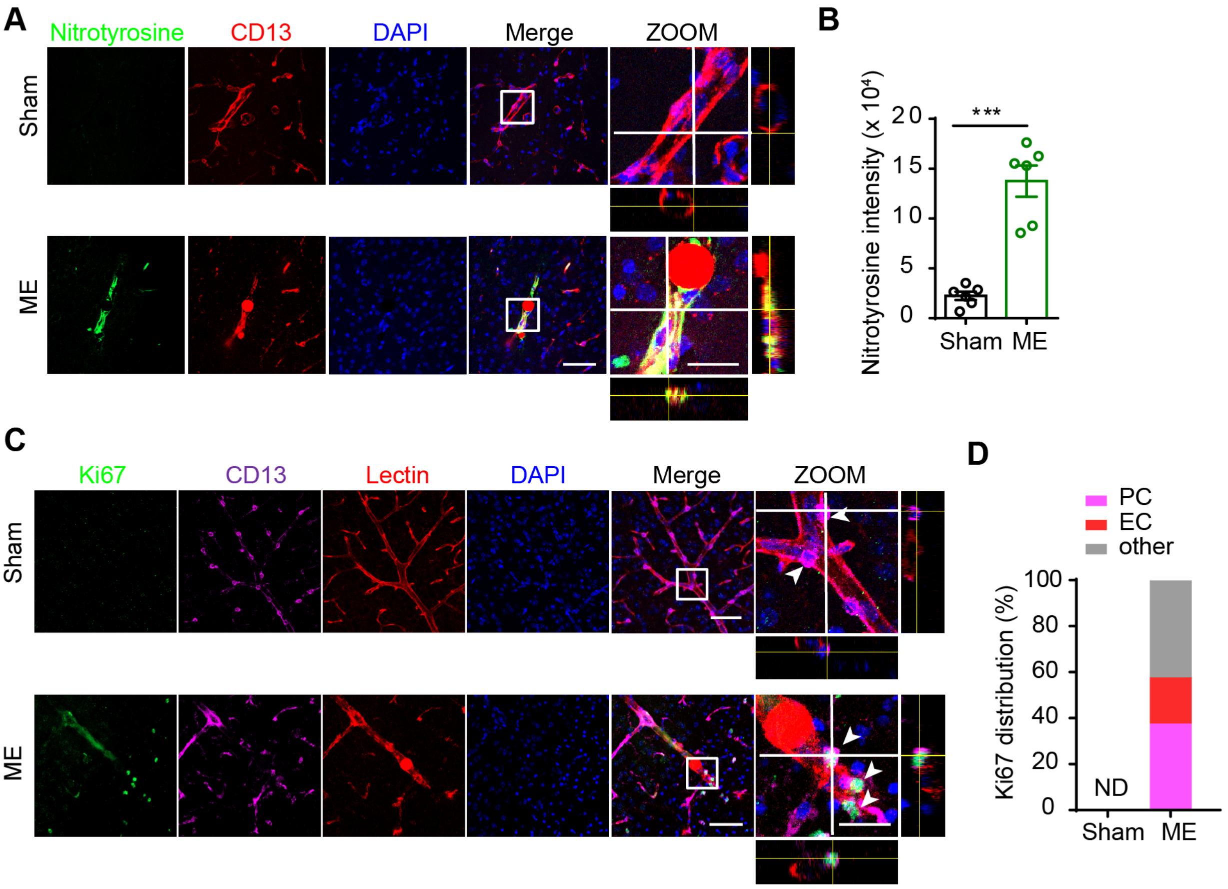

Figure S1. Nitrosative stress observed in the vessel after ME induction. (**A**) Maximum intensity projections of confocal images from brain sections stained for Nitrotyrosine (green), CD13 (red) and DAPI (blue, nuclei) at 72 h following ME. The images on the right are magnifications of the boxed regions in the images. Scale bar: 50 μm, magnified images, 20 μm. (**B**) Signal intensities of Nitrotyrosine staining quantified from **A** (dot plot, Sham: n = 6; ME: n = 6). (**C**) Maximum intensity projections of confocal images from brain sections stained for Ki67 (green), CD13 (magenta), Lectin (red) and DAPI (blue, nuclei) at 72 h following ME. The images on the right are magnifications of the boxed regions in the images. Scale bar: 50 μm, magnified images, 20 μm. The arrowheads indicate pericytes. (**D**) Quantification of

the percentage of Ki67 in pericytes, endothelial cells and other cells (Sham: n = 6; ME: n = 6). ***P < 0.001 vs. control. The data are presented as the mean ± s.e.m. Statistical significance was determined by unpaired Student's t-test. ND, not detected.

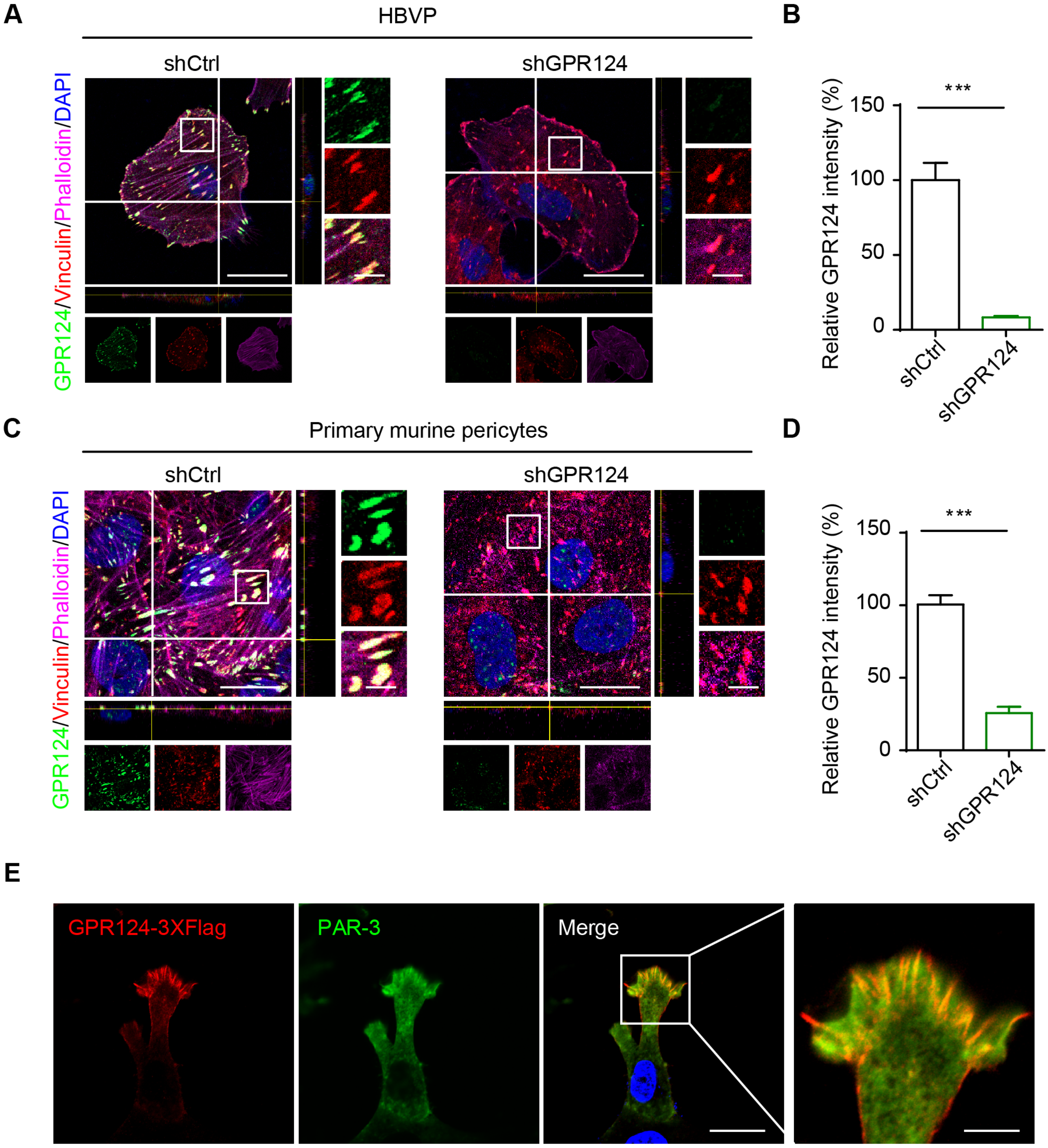

Figure S2. Representative images for knockdown and overexpression of GPR124 in pericytes. (**A**) Knockdown of GPR124 expression in HBVPs infected with either shCtrl or shGPR124 lentiviruses for 72 h. Cells were stained with GPR124 (red), Vinculin (green), Phalloidin (magenta) and DAPI (blue, nuclei). Scale bar: 20 μm (right), magnified images, 5 μm. (**B**) Signal intensities of GPR124 staining quantified from **A** (shCtrl: n = 20; shGPR124: n = 22). (**C**) Knockdown of GPR124 expression in primary mouse brain pericytes infected with either shCtrl or shGPR124 lentiviruses for 72 h. Cells were stained with GPR124 (red), Vinculin (green), Phalloidin (magenta) and DAPI (blue, nuclei). Scale bar: 20 μm (right), magnified images, 5 μm. (**D**) Signal intensities of GPR124 staining quantified from **C** (shCtrl: n = 21; shGPR124: n = 22). (**E**) Confocal images of Flag (red), PAR-3 (green) and DAPI (blue, nuclei) staining in the bodies of HBVPs showing the colocalization of GPR124 and polarization protein PAR-3 in the movement of the front tip of the cell. Scale bar: 20 μm (left) and 5 μm (right). ****P* < 0.001 vs. control. The data are presented as the mean ± s.e.m. Statistical significance was determined by unpaired Student's t-test.

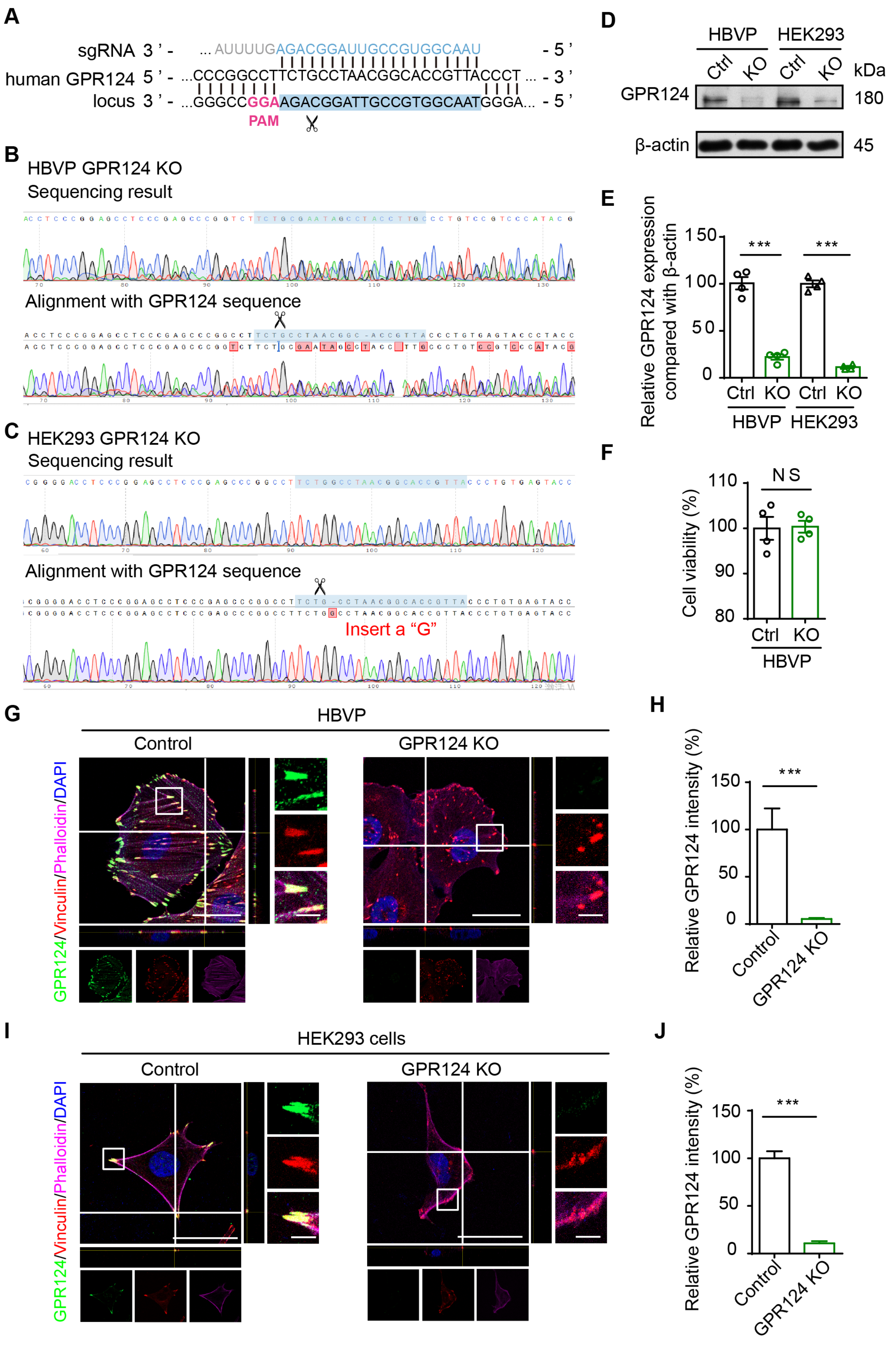

A
sgRNA 3'- ...AUUUUGAGACGGAUUGCCGUGGCAAU - 5'
human GPR124 5'- ...CCCGGCCTTCTGCCTAACGGCACCGTTACCCT ... - 3'
locus 3'- ...GGGCCGGAAGACGGATTGCCGTGGCAATGGGA... - 5'
PAM
B
HBVP GPR124 KO
Sequencing result
Alignment with GPR124 sequence
C
HEK293 GPR124 KO
Sequencing result
Alignment with GPR124 sequence
Insert a "G"
D
HBVP
HEK293
Ctrl
KO
Ctrl
KO
kDa
GPR124
180
β-actin
45
E
Relative GPR124 expression compared with β-actin
150
100
50
0
***
***
Ctrl
KO
Ctrl
KO
HBVP
HEK293
F
Cell viability (%)
110
100
90
80
NS
Ctrl
KO
HBVP
G
HBVP
Control
GPR124 KO
GPR124/Vinculin/Phalloidin/DAPI
H
Relative GPR124 intensity (%)
150
100
50
0
***
Control
GPR124 KO
I
HEK293 cells
Control
GPR124 KO
GPR124/Vinculin/Phalloidin/DAPI
J
Relative GPR124 intensity (%)
150
100
50
0
***
Control
GPR124 KO

Figure S3. CRISPR-Cas9-mediated GPR124 deletion in HEK293 cells and HBVPs. (**A**) Schematic of the Cas9-sgRNA-targeting sites in mutant GPR124 gene. Blue lines label the sgRNA-targeting sequences. (**B, C**) DNA sequence of PCR products amplified from the GPR124 gene of HBVPs (**B**) or HEK293 cells (**C**) aligns to the reference sequence. A "G" was inserted in the sequence of the GPR124 gene in HEK293 cell individual clones. Insertions are indicated with red labels. Lots of overlapped peaks were present in the sequences of PCR products amplified from the GPR124 gene in HBVP multiple clones. (**D**) Immunoblot analysis of GPR124 proteins level in Ctrl-HBVPs, GPR124 KO-HBVPs, Ctrl-HEK293 cells and GPR124 KO-HEK293 cells to confirm depletion of the corresponding proteins. (**E**) Quantitative analyses of the results as shown in **D** and presented in the bar graph as the densitometry ratio of the control from four independent experiments. (**F**) Cell viability was measured by the CCK8 assay in Ctrl-HBVPs and GPR124 KO-HBVPs. The data within each group were normalized to the viability of Ctrl ($n = 4$). (**G-J**) Confocal images of GPR124 (green), Vinculin (red), Phalloidin (magenta) and DAPI (blue, nuclei) staining in Ctrl-HBVPs and GPR124 KO-HBVPs (**G**), Ctrl-HEK293 cells and GPR124 KO-HEK293 cells (**I**). Scale bar: 40 μm, magnified images, 5 μm. **H** and **J** showed that signal intensities of GPR124 staining quantified from **G** and **I**, respectively (**H**, $n = 12$; **J**, $n = 14$). $^{***}P < 0.001$ vs. control. The data are presented as the mean ± s.e.m. Statistical significance was determined by unpaired Student's t-test. NS, not statistically significant.

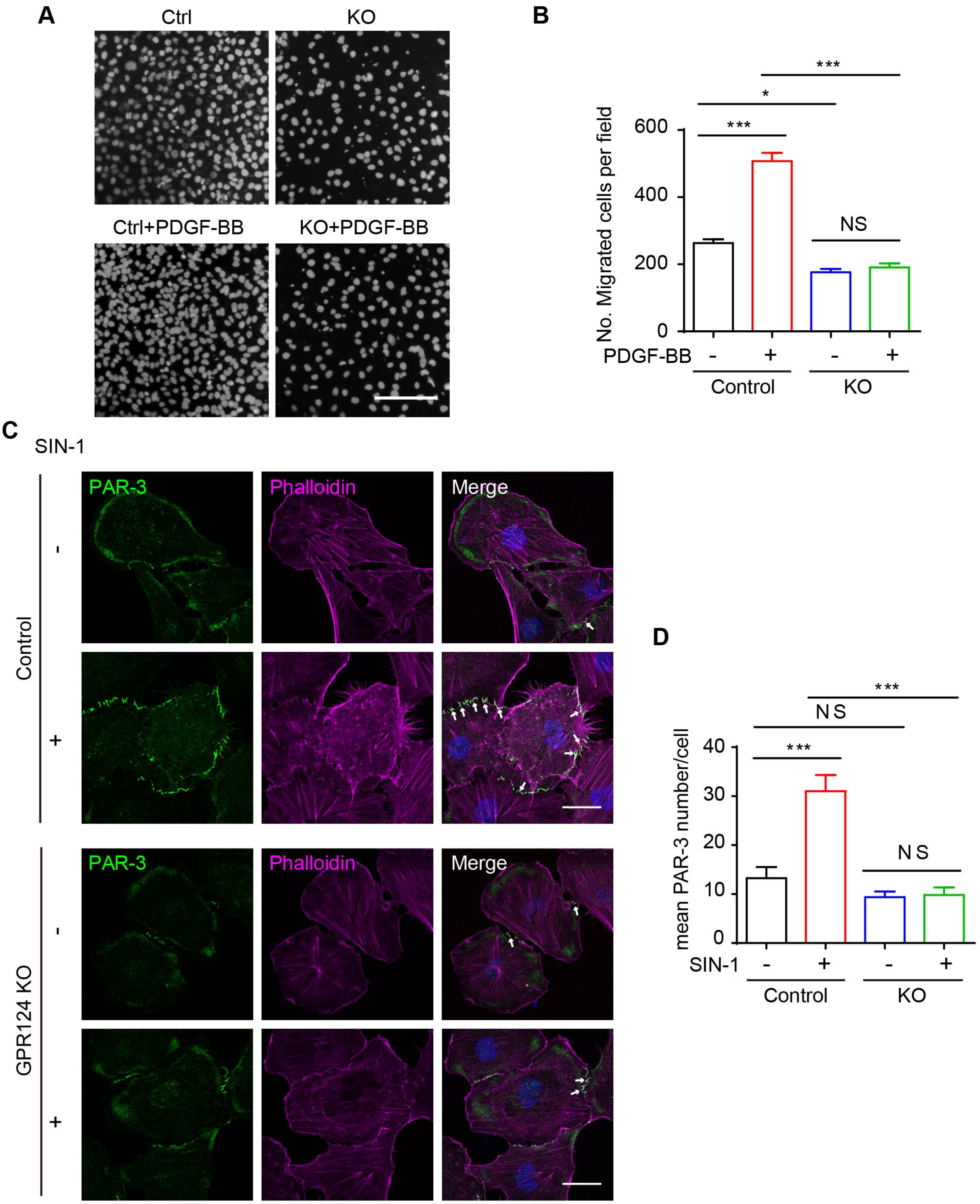

A
Ctrl
KO
Ctrl+PDGF-BB
KO+PDGF-BB
B
***
*
***
NS
600
400
200
0
No. Migrated cells per field
PDGF-BB
-
+
-
+
Control
KO
C
SIN-1
PAR-3
Phalloidin
Merge
Control
-
+
GPR124 KO
D
***
N S
***
N S
40
30
20
10
0
mean PAR-3 number/cell
SIN-1
Control
KO

Figure S4. GPR124 regulates cell polarity in HBVPs upon nitrosative stress. (**A**) Role of GPR124 on the migratory response of HBVPs to PDGF-BB was detected by a transwell invasion assay. PDGF-BB, 100 ng/mL. Scale bar: 200 μm. (**B**) Quantification of the number of migrated cell per field. Data are expressed as mean ± s.e.m. from three independent experiments (n = 3). (**C**) Confocal images of PAR-3 (green), Phalloidin (magenta) and DAPI (blue, nuclei) staining in the normal HBVPs or GPR124-KO HBVPs. The cells were seeded on glass-bottom 24-well plates overnight and then stimulated with or without SIN-1 (0.5 mM, 12 h). The distribution of PAR-3 at the cell edges is indicated by arrows. Scale bar: 20 μm. (**D**) Quantification of PAR-3-labeled filopodia for mean filopodia number per cell (n = 20). *$P < 0.05$ and ***$P < 0.001$ vs. control. The data are presented as the mean ± s.e.m. Statistical significance was determined by one-way ANOVA with Dunnett's multiple comparisons test. NS, not statistically significant.

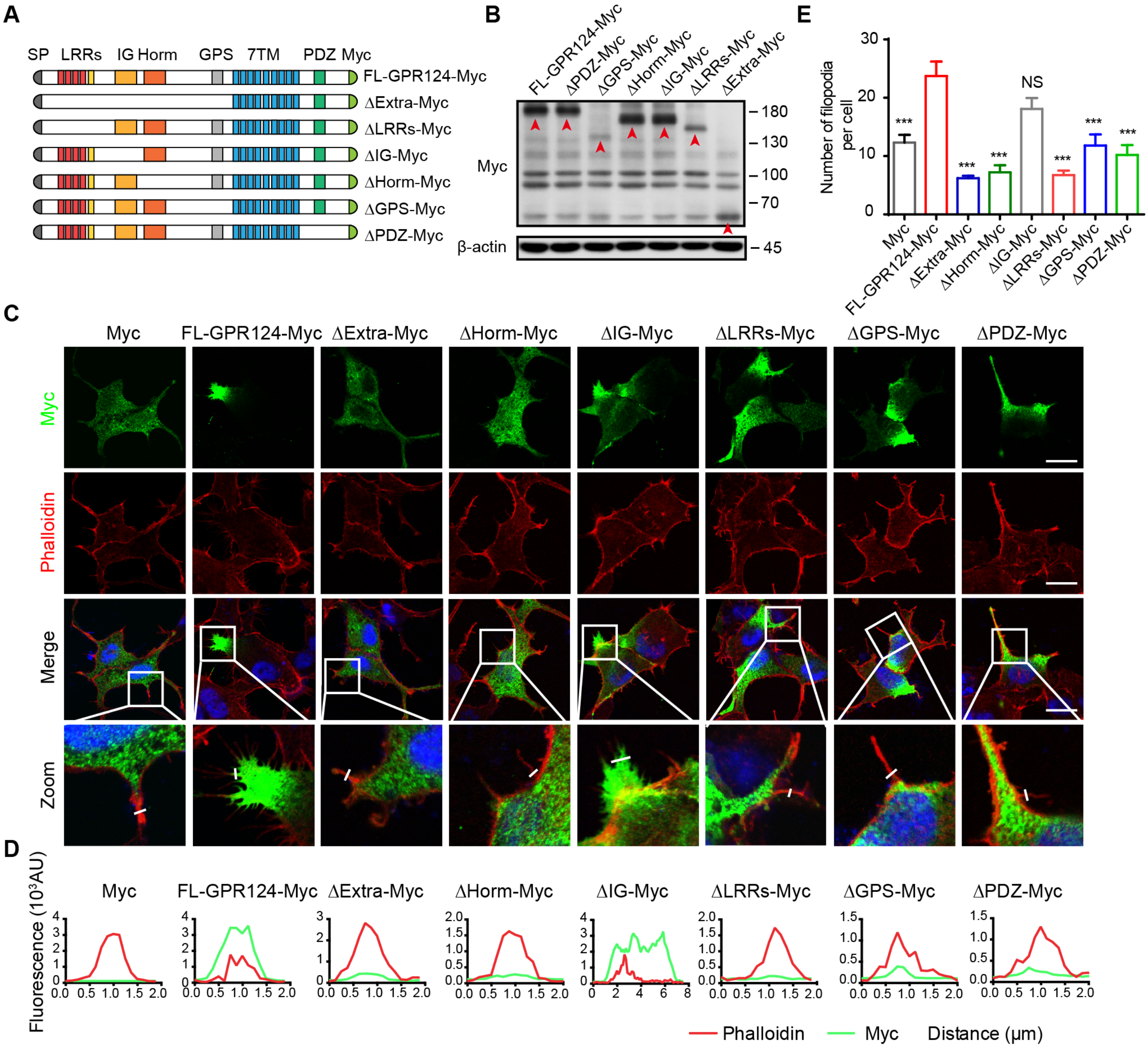

Figure S5. Essential domain of GPR124 required for SIN-1-induced migration in HEK293 cells. (**A**) Schematic diagram of full-length (FL) GPR124 and the N-terminal or C-terminal deletion mutants. (**B**) Immunoblot analysis of FL-GPR124-Myc or Myc-tagged GPR124 truncation mutants produced by transfection of HBVPs. Actin serves as a loading control. Molecular weight markers are indicated at the left. (**C**) Confocal images of Myc (green), phalloidin (red) and

DAPI (blue) stained HEK293 cells. GPR124-KO HEK293 cells transfected with FL-GPR124-Myc or Myc-tagged GPR124 truncation mutants as indicated. Cells were plated on coverslips 12 h before plasmid transfection and then incubated with SIN-1 (0.5 mM, 4 h). Single channels are shown in rows 1 and 2. The 4th row shows higher-magnification images of the boxed regions in the 3rd row. Scale bar: 50 μm (1st, 2nd and 3rd rows) and 10 μm (4th row). (**D**) Plots of the fluorescence intensity in arbitrary units (AU) along the solid lines indicated in the high-magnification images in **C**. (**E**) Quantification of the number of filopodia per cell (n = 10, 11, 19, 19, 12, 11, 15, 11). ***P < 0.001 vs. FL-GPR124-Myc. The data are presented as the mean ± s.e.m. Statistical significance was determined by unpaired Student's t-test. NS, not statistically significant.

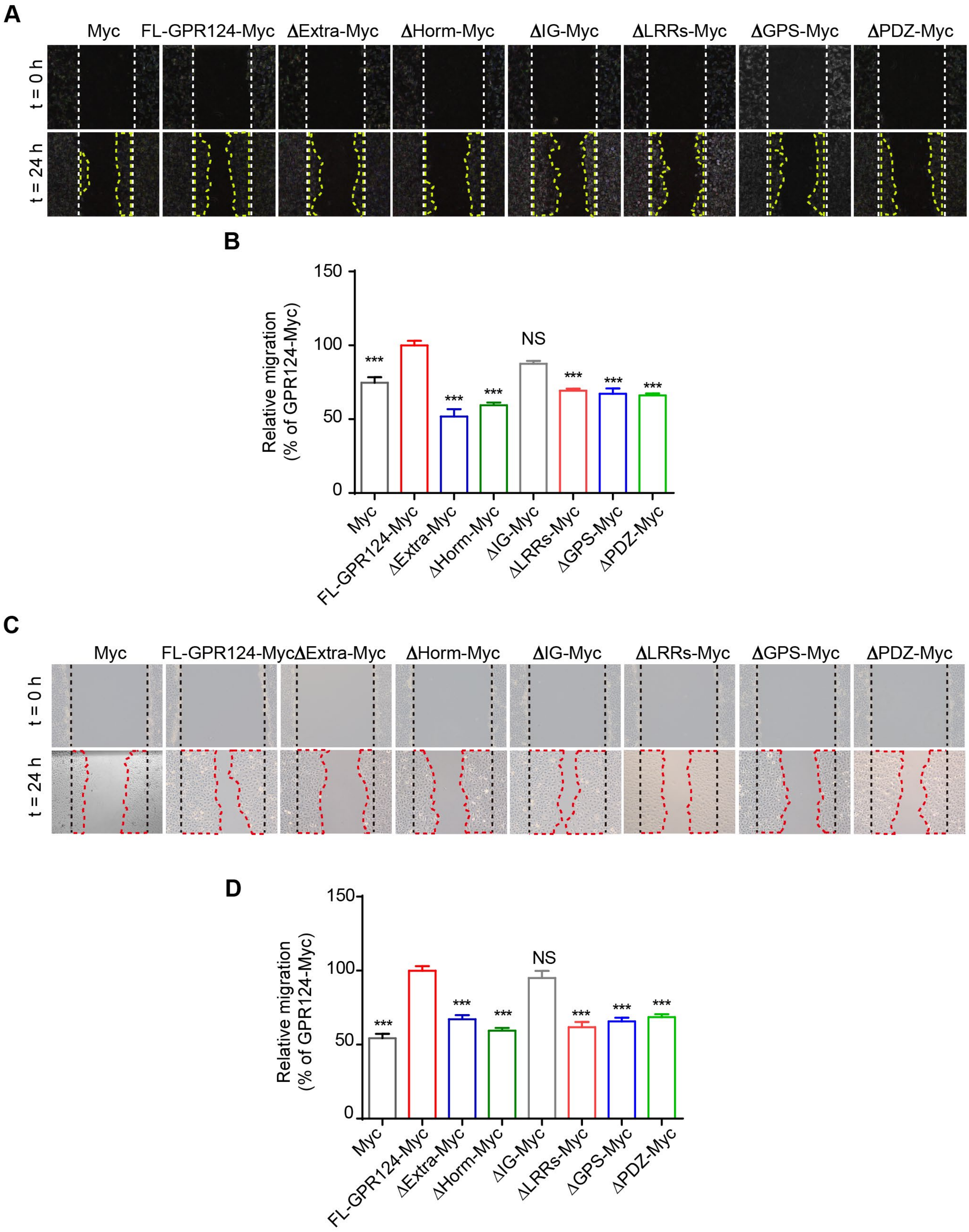

Figure S6. GPR124 regulates HBVPs migration through Horm domain. (A)

Representative images of GPR124-KO HEK293 cells transfected with FL-GPR124-Myc or Myc-tagged GPR124 truncation mutants at the 0 h and 24 h of the wound healing assay. The dashed lines (white) indicate the edges of the wound immediately after scratch. The dashed lines (yellow) outline the area of cell migration. (**B**) Quantification of relative migration by control and GPR124 truncates in a wound healing assay after 24 h (normalized to FL-GPR124-Myc values; n = 4 in each group). (**C**) Representative images of GPR124-KO HEK293 cells transfected with FL-GPR124-Myc or Myc-tagged GPR124 truncation mutants at the 0 h and 24 h of the wound healing assay. The dashed lines (black) indicate the edges of the wound immediately after scratch. The dashed lines (red) outline the area of cell migration. (**D**) Quantification of relative migration by control and GPR124 truncates in a wound healing assay after 24 h (normalized to FL-GPR124-Myc values; n = 4 in each group). ***$P < 0.001$ vs. FL-GPR124-Myc. The data are presented as the mean ± s.e.m. Statistical significance was determined by unpaired Student's t-test. NS, not statistically significant.

Table S1

Table. Mean, s.e.m. and statistical analyses for data presented in Figures 1-8, Related to Figures 1-8.						
**FIGURE NUMBER**	**TEST USED**	**n**		**DESCRIPTIVE STATS (AVERAGE, VARIANCE)**	**P VALUE**	**DEGREES OF FREEDOM & F/t/z/R/ETC VALUE**
Figure	WHICH TEST?	EXACT VALUE	DEFINED?	REPORTED?	EXACT VALUE	VALUE
**1B**	unpaired two-tailed Student's t-test	4, 4	Mice	error bars are mean +/- SEM	P = 0.0015	
**1C**	unpaired two-tailed Student's t-test	4, 4	Mice	error bars are mean +/- SEM	P = 0.0089	
**1D**	unpaired two-tailed Student's t-test	4, 4	Mice	error bars are mean +/- SEM	P = 0.5816	
**1E**	unpaired two-tailed Student's t-test	4, 4	Mice	error bars are mean +/- SEM	P = 0.0575	
**1I**	unpaired two-tailed Student's t-test	6, 6	Mice	error bars are mean +/- SEM	P = 0.0034	
**4C**	unpaired two-tailed Student's t-test	4, 4	experiments	error bars are mean +/- SEM	P < 0.0001	
**4F**	unpaired two-tailed Student's t-test	4, 4, 4	experiments	error bars are mean +/- SEM	P < 0.0001; P < 0.0001	
**4G**	unpaired two-tailed Student's t-test	4, 4	experiments	error bars are mean +/- SEM	P = 0.3079	
**4I**	unpaired two-tailed Student's t-test	4, 4	experiments	error bars are mean +/- SEM	P < 0.0001; P < 0.0001	
**4M**	one-way ANOVA followed by a	4, 4,4,4,4,4	experiments	error bars are mean +/- SEM	P < 0.0001	F (5, 18) = 10.39

	Dunnett's Multiple Comparisons Test					
**4N**	one-way ANOVA followed by a Dunnett's Multiple Comparisons Test	3, 3,3,3,3,3	experiments	error bars are mean +/- SEM	P = 0.0002	F (5, 12) = 12.18
**5B**	one-way ANOVA followed by a Dunnett's Multiple Comparisons Test	9, 18, 9, 9	cell	error bars are mean +/- SEM	P < 0.0001	F (3, 41) = 48.8
**5C**	one-way ANOVA followed by a Dunnett's Multiple Comparisons Test	11, 16, 11, 10	cell	error bars are mean +/- SEM	P < 0.0001	F (3, 44) = 17.79
**5E**	one-way ANOVA followed by a Dunnett's Multiple Comparisons Test	12, 12, 12, 12	cell	error bars are mean +/- SEM	P < 0.0001	F (3, 44) = 69.8
**5F**	one-way ANOVA followed by a Dunnett's Multiple Comparisons Test	12, 30, 12, 11	cell	error bars are mean +/- SEM	P = 0.0014	F (3, 64) = 5.804
**5H**	one-way ANOVA followed by a Dunnett's Multiple	18, 15, 12, 9	cell	error bars are mean +/- SEM	P = 0.0415	F (3, 50) = 2.999

	Comparisons Test					
**5I**	one-way ANOVA followed by a Dunnett's Multiple Comparisons Test	12, 17, 11, 19	cell	error bars are mean +/- SEM	P < 0.0001	F (3, 54) = 8.615
**7I**	unpaired two-tailed Student’s t-test	26, 26, 26, 26, 26, 26, 26, 26	cell	error bars are mean +/- SEM		
				Myc vs. FL-GPR124-Myc<br>ΔExtra-Myc vs. FL-GPR124-Myc<br>ΔHorm-Myc vs. FL-GPR124-Myc<br>ΔIg-Myc vs. FL-GPR124-Myc<br>ΔLRRMyc vs. FL-GPR124-Myc<br>ΔGPS-Myc vs. FL-GPR124-Myc<br>ΔPDZ-Myc vs. FL-GPR124-Myc	P < 0.0001<br>P < 0.0001<br>P < 0.0001<br>P = 0.3027<br>P = 0.1451<br>P = 0.7321<br>P = 0.6139	
**7J**	unpaired two-tailed Student’s t-test	20, 20, 20, 20, 20, 20, 20, 20	cell	error bars are mean +/- SEM		
				Myc vs. FL-GPR124-Myc<br>ΔExtra-Myc vs. FL-GPR124-Myc<br>ΔHorm-Myc vs. FL-GPR124-Myc<br>ΔIg-Myc vs. FL-GPR124-Myc<br>ΔLRRMyc vs. FL-GPR124-Myc<br>ΔGPS-Myc vs. FL-GPR124-Myc<br>ΔPDZ-Myc vs. FL-GPR124-Myc	P < 0.0001<br>P < 0.0001<br>P < 0.0001<br>P = 0.4953<br>P < 0.0001<br>P < 0.0001<br>P < 0.0001	

**8B**	one-way ANOVA followed by a Dunnett's Multiple Comparisons Test	3, 3, 3, 3	experiments	error bars are mean +/- SEM	P = 0.0008	F (3, 8) = 17.04
**Table. Mean, s.e.m. and statistical analyses for data presented in Supplementary Figures 1-6, Related to Supplementary Figures 1-6.**						
**FIGURE NUMBER**	**TEST USED**	**n**		**DESCRIPTIVE STATS (AVERAGE, VARIANCE)**	**P VALUE**	**DEGREES OF FREEDOM & F/t/z/R/ETC VALUE**
Figure	WHICH TEST?	EXACT VALUE	DEFINED?	REPORTED?	EXACT VALUE	VALUE
**S1B**	unpaired two-tailed Student's t-test	6, 6	mice	error bars are mean +/- SEM	P < 0.0001	
**S2B**	unpaired two-tailed Student's t-test	20, 22	cell	error bars are mean +/- SEM	P = 0.0002	
**S2D**	unpaired two-tailed Student's t-test	21, 22	cell	error bars are mean +/- SEM	P < 0.0001	
**S3E**	unpaired two-tailed Student's t-test	4, 4, 4, 4	experiments	error bars are mean +/- SEM	P < 0.0001; P < 0.0001	
**S3F**	unpaired two-tailed Student's t-test	4, 4	experiments	error bars are mean +/- SEM	P = 0.9010	
**S3H**	unpaired two-tailed Student's t-test	12, 12	cell	error bars are mean +/- SEM	P < 0.0001	
**S3J**	unpaired two-tailed Student's t-test	14, 14	cell	error bars are mean +/- SEM	P < 0.0001	
**S4B**	one-way ANOVA followed by a	3, 3, 4, 3	experiments	error bars are mean +/- SEM	P < 0.0001	F (3, 9) = 99.45

	Dunnett's Multiple Comparisons Test					
**S4D**	one-way ANOVA followed by a Dunnett's Multiple Comparisons Test	20, 20, 20, 20	cell	error bars are mean +/- SEM	P < 0.0001	F (3, 76) = 50.29
**S5E**	unpaired two-tailed Student's t-test	10, 11, 19, 19, 12, 11, 15, 11	cell	error bars are mean +/- SEM		
				Myc vs. FL-GPR124-Myc<br>ΔExtra-Myc vs. FL-GPR124-Myc<br>ΔHorm-Myc vs. FL-GPR124-Myc<br>ΔIg-Myc vs. FL-GPR124-Myc<br>ΔLRRMyc vs. FL-GPR124-Myc<br>ΔGPS-Myc vs. FL-GPR124-Myc<br>ΔPDZ-Myc vs. FL-GPR124-Myc	P = 0.0009<br>P < 0.0001<br>P < 0.0001<br>P = 0.0828<br>P < 0.0001<br>P = 0.0007<br>P = 0.0002	
**S6B**	unpaired two-tailed Student's t-test	4, 4, 4, 4, 4, 4, 4, 4	experiments	error bars are mean +/- SEM		
				Myc vs. FL-GPR124-Myc<br>ΔExtra-Myc vs. FL-GPR124-Myc<br>ΔHorm-Myc vs. FL-GPR124-Myc<br>ΔIg-Myc vs.	P < 0.0001<br>P = 0.0002<br>P < 0.0001<br>P =	

				FL-GPR124-Myc<br>ΔLRRMyc vs.<br>FL-GPR124-Myc<br>ΔGPS-Myc vs.<br>FL-GPR124-Myc<br>ΔPDZ-Myc vs.<br>FL-GPR124-Myc	0.4192<br>P <<br>0.0001<br>P =<br>0.0001<br>P =<br>0.0001	
**S6D**	unpaired two-tailed Student's t-test	4, 4, 4, 4, 4, 4, 4, 4	experiments	error bars are mean +/- SEM		
				Myc vs.<br>FL-GPR124-Myc<br>ΔExtra-Myc vs.<br>FL-GPR124-Myc<br>ΔHorm-Myc vs.<br>FL-GPR124-Myc<br>ΔIg-Myc vs.<br>FL-GPR124-Myc<br>ΔLRRMyc vs.<br>FL-GPR124-Myc<br>ΔGPS-Myc vs.<br>FL-GPR124-Myc<br>ΔPDZ-Myc vs.<br>FL-GPR124-Myc	P <<br>0.0001<br>P =<br>0.0002<br>P <<br>0.0001<br>P =<br>0.2141<br>P <<br>0.0001<br>P =<br>0.0005<br>P <<br>0.0001	
